# Adipokines and Inflammation: Focus on Cardiovascular Diseases

**DOI:** 10.3390/ijms21207711

**Published:** 2020-10-18

**Authors:** Sandra Feijóo-Bandín, Alana Aragón-Herrera, Sandra Moraña-Fernández, Laura Anido-Varela, Estefanía Tarazón, Esther Roselló-Lletí, Manuel Portolés, Isabel Moscoso, Oreste Gualillo, José Ramón González-Juanatey, Francisca Lago

**Affiliations:** 1 Cellular and Molecular Cardiology Unit and Department of Cardiology, Institute of Biomedical Research of Santiago de Compostela (IDIS-SERGAS), Travesía Choupana s/n, 15706 Santiago de Compostela, Spain; alana.aragon.herrera@sergas.es (A.A.-H.); sandra.moranha@gmail.com (S.M.-F.); laura.anidovarela67@gmail.com (L.A.-V.); jose.ramon.gonzalez.juanatey@sergas.es (J.R.G.-J.); francisca.lago.paz@sergas.es (F.L.); 2 CIBERCV, Institute of Health Carlos III, C/Monforte de Lemos 3-5, Pabellón 11, Planta 0, 28029 Madrid, Spain; tarazon_est@gva.es (E.T.); esther_rosello_lleti@hotmail.com (E.R.-L.); portoles_man@gva.es (M.P.); imosgal@gmail.com (I.M.); 3 Cardiology Group, Center for Research in Molecular Medicine and Chronic Diseases (CIMUS) and Institute of Biomedical Research of Santiago de Compostela (IDIS-SERGAS), Av. Barcelona, Campus Vida, Universidade de Santiago de Compostela, 15782 Santiago de Compostela, Spain; 4 Cardiocirculatory Unit, Health Research Institute of La Fe University Hospital, Avinguda de Fernando Abril Martorell 106, 46026 Valencia, Spain; 5 SERGAS (Servizo Galego de Saude): Área Sanitaria de Santiago de Compostela e Barbanza. Santiago University Clinical Hospital, Research Laboratory 9, 15706 Santiago de Compostela, Spain and IDIS (Instituto de Investigación Sanitaria de Santiago), The NEIRID Group (NeuroEndocrine Interactions in Rheumatic and Inflammatory Diseases) C027, 15706 Santiago de Compostela, Spain; oreste.gualillo@sergas.es

**Keywords:** adipokines, inflammation, cardiovascular diseases

## Abstract

It is well established that adipose tissue, apart from its energy storage function, acts as an endocrine organ that produces and secretes a number of bioactive substances, including hormones commonly known as adipokines. Obesity is a major risk factor for the development of cardiovascular diseases, mainly due to a low grade of inflammation and the excessive fat accumulation produced in this state. The adipose tissue dysfunction in obesity leads to an aberrant release of adipokines, some of them with direct cardiovascular and inflammatory regulatory functions. Inflammation is a common link between obesity and cardiovascular diseases, so this review will summarise the role of the main adipokines implicated in the regulation of the inflammatory processes occurring under the scenario of cardiovascular diseases.

## 1. Introduction

Despite the knowledge and the numerous preventive and therapeutic measures available nowadays, cardiovascular diseases (CVDs) are the leading cause of death globally, accounting for an estimated 31% of all deaths worldwide [1]. Among the risk factors triggering the development of CVDs, obesity and adipose tissue accumulation are the ones that attract the most focus of the scientific community as a potential niche for the search of new therapeutic targets that could help prevent or alleviate the pathogenesis of CVDs. 

White adipose tissue (WAT) is a specialised organ with functions not only in the storage and release of energy substrates for systemic metabolism regulation but also in the production of different secreted factors that act as paracrine and endocrine regulators [2]. These secretions include a variety of chemical agents (including cytokines and chemokines), which interact with other adipocytes, immune cells, and somatic cells, with some of them being secreted by non-adipocyte cells like fibroblasts, vascular cells, etc. and others secreted from adipocytes and thus classified as adipokines [3]. Adipokines act as autocrine/paracrine/endocrine mediators, mainly regulating appetite, metabolism, immunity, behaviour, cardiovascular function, and reproduction [2]. Therefore, adipose tissue is at the same time responsive to and responsible for a wide variety of hormonal, inflammatory, and metabolic interactions with other organs, so the proper functioning of the adipose tissue is essential to maintaining good health. 

Obesity is recognised as increasing CVDs’ morbidity and mortality through different mechanisms, including the hemodynamic adaptation of the cardiovascular system due to the increase of body weight; the development of co-morbidities associated with both obesity and CVDs, such as type 2 diabetes mellitus (T2DM), insulin resistance, hypertension or dyslipidaemia; and the imbalance in the production of adipokines by the adipose tissue [4]. However, not all obese subjects have the same probability of developing co-morbidities or the same mortality risk, and adipokines are some of the players responsible for this situation. 

Subcutaneous adipose tissue (SAT) is the largest WAT depot in lean, healthy subjects, representing about 80% of all adipose tissue, and thus its role as an energy store is more important than any other fat depot [5]. In response to a positive energy balance, the adipose tissue undergoes reorganization by changing the number and size of mature adipocytes. Hypertrophic adipocytes secrete adipokines, which recruit preadipocytes and promote their differentiation into mature adipocytes, a process commonly known as adipose tissue remodelling [6]. When SAT expansion due to overnutrition is achieved by adipose tissue remodelling, the WAT is able to keep its regulatory functions, and this situation is called “metabolically healthy obesity” [6]. Metabolically healthy obese subjects do not have a higher mortality risk and do not suffer from metabolic abnormalities like dyslipidaemia, insulin resistance, hypertension, or a pro-inflammatory profile [7]. Although it is not fully understood, some of the mechanisms postulated to explain metabolically healthy obesity include preserved insulin sensitivity, a higher fat accumulation in SAT than in visceral and ectopic depots, normal adipose tissue function, and normal adipokine secretion [7]. 

**Figure 1:**
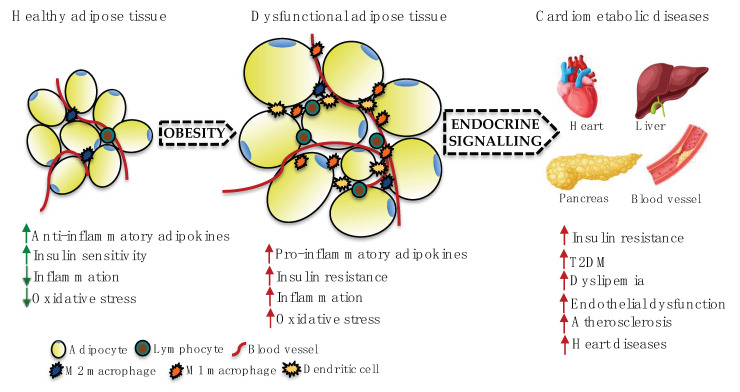
Obesity, inflammation, and cardiometabolic diseases (CMDs). In the obese adipose tissue, the increased size of adipocytes and the infiltration of immune cells produce a deregulation of its physiological function. Dysfunctional adipose tissue leads to a chronic low-grade inflammation state due to altered production of adipokines/cytokines, which are secreted into the bloodstream and reach other tissues, affecting their biology in a paracrine or endocrine manner. Many of the known circulating pro-inflammatory molecules that directly participate in the development of CMDs are released from adipocytes/adipose tissue, linking obesity to a higher risk of developing CVDs. Green arrows: beneficial effect, red arrows: detrimental effect.

On the other hand, when SAT does not expand properly to store the energy excess, it becomes dysfunctional, leading to fat accumulation in ectopic tissues, including the liver, pancreas, skeletal muscle, and heart, as well as in the visceral cavity, predisposing to cardiometabolic deregulation [6]. Dysfunctional adipose tissue becomes insulin-resistant and produces pro-inflammatory cytokines and extracellular matrix proteins that promote infiltration and activation of immune cells, creating an optimal environment for inflammation [6,8]. At the same time, activated immune cells secrete cytokines that modulate adipocyte function, differentiation, and adipokine secretion, favouring the increase of pro-inflammatory adipokine expression in the adipose tissue such as leptin or resistin [8,9]. Taken together, this situation affects systemic metabolic homeostasis and inflammation in such a way that the different fat depots contribute to the metabolic impairment and chronic low-grade inflammation, and to the development of obesity-associated co-morbidities such as insulin resistance, T2DM, metabolic syndrome, and cardiovascular diseases (Figure 1) [10,11]. 

In order to prevent and ameliorate the development of cardiometabolic diseases (CMDs), it is of great importance to know the pathophysiological mechanisms that link obesity and CMDs. In this review, we will focus on the role of some traditional and novel adipokines implicated in inflammatory processes that mediate the development of CMDs, aiming to provide updated insights into the possible therapeutic regulation of these molecules. 

## 2. Adipose Tissue Dysfunction and CVDs

The body’s distribution of adipose tissue is key in the development of obesity co-morbidities: the altered secretion of adipose-derived factors due to the dysfunction of the adipose tissue affects not only the adipose tissue itself in an autocrine/paracrine manner but also other metabolic and non-metabolic organs, contributing to obesity-related cardiometabolic imbalance and CVDs [12,13]. Adipose tissue secretions from the different fat depots can reach cardiovascular sites, such as the heart or arteries, and regulate their biology in an endocrine manner. In particular, adipose depots located in the cardiovascular system, like perivascular and epicardial adipose tissues, can exert direct effects on the adjacent vascular wall or myocardium, respectively, through the paracrine release of adipokines, which can also reach the lumen of the adjacent blood vessels and travel downstream, affecting the vascular tone, inflammation, endothelial function, or vascular redox state of the entire vascular beds in a vasocrine manner [14].

Under physiological conditions, adipocytes predominantly secrete anti-inflammatory adipokines such as adiponectin, transforming growth factor-β (TGF-β), interleukin 10 (IL-10), or nitric oxide (NO), which promote insulin sensitivity and exert cardioprotective and anti-atherogenic effects. However, in pathological conditions such as obesity, dysfunctional adipocytes mostly produce and release pro-inflammatory adipokines such as leptin, tumour necrosis factor-α (TNFα), interleukin 6 (IL-6), interleukin 18 (IL-18), or resistin, which have atherogenic effects [4]. In general terms, it is established that visceral obesity is strongly related to the development of cardiovascular risk factors as insulin resistance, atherogenic dyslipidemia, or hypertension, and with the production of pro-inflammatory adipokines, while subcutaneous fat deposition has a protective effect on CVD risk and mainly secretes anti-inflammatory adipokines [15,16,17].

Regarding ectopic fat accumulation, adipose depots located in major glucose-regulatory organs such as the liver, skeletal muscle, and pancreas usually deregulate insulin signalling, promoting insulin resistance and increasing the risk for T2DM and CVDs [5,8]. Although the initial pathogenic processes that lead to the development of insulin resistance in obesity are not fully understood, it seems clear that the inflammatory response in the adipose tissue and the imbalance in adipokine secretion are some of the triggers [8]. Epicardial adipose tissue (EAT) dysfunction is associated with the release of pro-inflammatory adipokines and the infiltration of immune cells and with the decrease in the production of anti-inflammatory adipokines, contributing to the development of metabolic syndrome [18]. EAT is tightly connected with the myocardium, sharing the same microcirculation vasculature and with the coronary arteries embedded in EAT [12]. Thus, due to its proximity to the heart, EAT deregulation can affect myocardial function by increasing cardiac lipid accumulation, insulin resistance, and fibrosis due to a decreased secretion of anti-inflammatory adipokines such as adiponectin and an increased secretion of pro-inflammatory adipokines like leptin, TNFα, IL-1β, IL-6, or resistin [12,19]. Moreover, it has been proposed that the adipokines released by EAT in pathological conditions have paracrine effects on cardiac electrical activity, affecting conductivity and promoting atrial fibrillation [20], and in coronary arteries, they cause atherosclerosis through the promotion of inflammation and immune cell infiltration [21,22]. Lately, EAT thickness has been considered a clinical biomarker that correlates to features of heart failure and metabolic syndrome [23,24,25,26,27]. Another fat depot that surrounds the coronary arteries is the perivascular adipose tissue (PVAT). With the exception of cerebral vessels, PVAT is found almost ubiquitously on vasculature throughout the body and is markedly increased in obesity [28]. Compared with lean PVAT, obese PVAT secretes more pro-inflammatory adipokines, including TNFα, leptin, IL-6, plasminogen activator, and resistin, which switch PVAT into a pro-inflammatory and pro-oxidative phenotype that promotes atherosclerotic plaque formation and instability, not only in coronary arteries but also in other blood vessels [28,29,30,31,32,33,34]. In addition, PVAT dysfunction has been related with the deregulation of blood vessels contractility, so the inflammation and oxidative stress abolish PVAT’s natural protective anti-contractile effect, contributing to the development of hypertension [35,36,37]. 

## 3. Role of Some Adipokines in Inflammatory Processes Associated with CVDs

### 3.1. Leptin

Leptin was the first adipokine discovered and is the most studied so far. Under physiological conditions, leptin mainly decreases appetite, increases energy expenditure, and regulates glucose homeostasis independently of insulin action through hypothalamic and sympathetic signalling. It also regulates cardiac and vascular function by a NO-dependent mechanism [38]. In obesity, circulating leptin levels are increased, as is its mRNA expression in adipocytes in obese patients [39]. However, despite hyperleptinemia, obese individuals are leptin-resistant, which means the failure of leptin to decrease appetite and promote energy expenditure [40]. Moreover, leptin resistance is also associated with the development of hypertension and insulin resistance [41]. Inflammation is one of the mechanisms thought to be responsible for leptin resistance [42]. 

Despite its positive physiological functions, leptin is traditionally considered a pro-inflammatory cytokine. It belongs to the family of long-chain helical cytokines, showing homology in its molecular structure with other cytokines like TNFα, IL-6, IL-12, or IL-15. It has been suggested that leptin could act as an acute-phase inflammatory protein, so its circulating concentrations are increased during sepsis and fever and its production can be stimulated by other inflammatory mediators such as TNFα and IL-1 [42,43]. Leptin has a direct role in the inflammatory response by activating monocytes, leukocytes, and macrophages to produce IL-6, TNFα, and IL-12, increasing the generation of reactive oxygen species (ROS) and migratory responses in monocytes and increasing the production of CC-chemokine ligands in macrophages [8,43,44]. 

Studies carried out in leptin-deficient/resistant murine animal models have shown that leptin participates in the regulation of cardiac metabolism, contraction, hypertrophy, and apoptosis, with some contradictory results but overall indicating that the deregulation of leptin signalling may have important implications in heart physiology [45]. In this line, leptin-deficient *ob/ob* and leptin-resistant *db/db* mice suffer from impaired cardiac function due to cardiac hypertrophy, inflammation, oxidative stress, and iron overload, an effect that is reverted by calorie restriction through the restoring of iron levels [46].

Hyperleptinemia is considered an independent risk factor for coronary artery disease (CAD) and a strong predictor of acute myocardial infarction (AMI) [47,48]. In mice with leptin overexpression, cardiac ischemia/reperfusion (I/R) leads to cardiomyocyte hypertrophy and fibrosis and worsens myocardial dysfunction [49]. Patients with myocardial infarction (MI) have increased circulating levels of leptin, which correlates with pro-inflammatory markers, suggesting that leptin is associated with the inflammatory response produced during an AMI [50,51,52]. Patients with CAD also have increased leptin circulating levels, along with other pro-inflammatory markers such as TNFα, C-reactive protein (CRP), or IL-6, as well as higher leptin expression in SAT, EAT, and PVAT, with leptin expression in EAT being considered as an independent risk factor for coronary atherosclerosis [53,54,55,56]. Increased circulating leptin levels in patients with CAD have been associated with short-term occurrence of cardiac remodelling, impaired diastolic function, cardiac heart failure, cardiac death, acute coronary syndrome, and stroke [57,58,59,60]. In women with CAD, increased circulating leptin levels can predict cardiovascular death and non-fatal MI [61,62]. Regarding the endothelium, several studies have suggested that leptin contributes to endothelial dysfunction [63,64,65]. It has been shown that leptin induces the expression of CRP, cellular adhesion molecules, and platelet tissue factor in human coronary endothelial cells, as well as oxidative stress in human umbilical vein endothelial cells (HUVECs), and inflammation, hypoxia, angiogenesis and fibrosis in human PVAT. This contributes to endothelial dysfunction and suggests a pro-atherogenic role of leptin under situations of hyperleptinemia [55,66,67,68,69]. 

### 3.2. Chemerin

Chemerin, also known as tazarotene-induced gene 2 (TIG2) or retinoic acid receptor responder 2 (RARRES2), is a chemoattractant protein for immune cells with regulatory functions in immunity, adipogenesis, metabolism, and inflammation [70,71]. It is synthesised as a prepropeptide, needing the N-terminal cleavage of a 20-amino acid signal peptide to be secreted as the 18 kDa inactive precursor prochemerin (chemerin163) [72]. Inactive prochemerin circulates in the bloodstream, but to exert its biological action, it needs to be activated by proteolytic cleavages at its C-terminus by extracellular proteases of the coagulation, fibrinolytic, and inflammatory cascades, leading to different chemerin forms [73]. According to the types of proteases that cleave the C-terminus from prochemerin, there can be three active chemerin products produced: chemerin156, chemerin157, and chemerin158, with different activity and affinity to the chemerin receptors chemokine-like receptor 1 (CMKLR1), G protein-coupled receptor 1 (GPR1), and C-C chemokine receptor-like 2 (CCRL2). Among these, CMKLR1 is the primary chemerin receptor [72] and the most studied, with wide expression in the body [74]. The CCRL2 receptor is considered to act as a binding site for chemerin to increase its concentration locally. In this way, prochemerin would bind to CCRL2, leaving its C-terminus exposed to be proteolytically processed into its active form and subsequently interact with adjacent cells expressing CMKLR1 receptors, which would trigger chemerin intracellular signalling [75,76,77]. The three chemerin receptors have in some cases the same tissue distribution, but they can also be differentially expressed. Therefore, according to the expression levels of the proteases that activate chemerin and the chemerin product that binds each type of receptor, chemerin signalling can vary in different tissues, even exerting contradictory effects [70,72,78,79]. 

Chemerin was first identified as a novel retinoid-responsive gene in psoriatic skin lesions [80]. In addition to skin, chemerin is widely expressed throughout the human body and is mainly produced by the liver, WAT, and placenta [70] but is also found in the brain, spinal cord, spleen, lymph node, thymus, stomach, small intestine, colon, kidneys, testis, ovary, pituitary, lungs, heart, skeletal muscle, pancreas, arteries, cartilage, or gingival tissues [74,81,82,83,84,85,86]. The adipose tissue production of chemerin and CMKLR1 receptor in humans and mice was described in 2007, when chemerin came to be considered a new adipokine [87]. Regarding the different adipose tissue depots, chemerin expression in humans has been described in SAT [88], visceral adipose tissue (VAT) [88,89,90], PVAT [83], and EAT [91,92]. Chemerin has been shown to promote the maturation of adipocytes, which increases the chemerin production by these cells, so adipocytes are both producers of and targets for chemerin [93]. In addition, chemerin has been suggested to participate in adipose tissue metabolism, with positive regulatory effects on insulin-stimulated glucose uptake by 3T3 adipocytes [93,94], although another work showed the contrary effect of chemerin treatment on 3T3 adipocytes [95]. In this line, different studies also showed a reduced insulin-stimulated glucose uptake by chemerin in human skeletal muscle [96], increased insulin resistance in rat cardiomyocytes [97]. In mouse models of obesity/diabetes, increased glucose intolerance, reduced serum insulin levels, and reduced tissue glucose uptake after chemerin administration were observed [98], despite the fact that some studies suggested that chemerin is necessary for the proper insulin production by the pancreas [99,100]. Although it seems clear that chemerin can affect insulin production and signalling, these contradictory results could be explained by the differential effect of chemerin in different tissues and pathophysiological conditions [99].

A number of studies have shown an association between increased circulating chemerin levels with obesity and T2DM in humans, correlating with markers of insulin resistance and inflammation [101,102,103,104], while exercise, diet intervention, and bariatric surgery decrease its levels [105,106,107,108]. Moreover, both chemerin and CMKLR1 are upregulated in adipose tissue from obese patients, where the pro-inflammatory cytokine TNFα increases chemerin mRNA expression in visceral adipocytes [109], which suggests that the low-grade inflammation observed in obesity could contribute to chemerin expression in the adipose tissue [110]. At the same time, chemerin can recruit circulating dendritic cells into visceral adipose tissue, contributing to inflammation and insulin resistance [111]. 

Although some studies describe anti-inflammatory actions of chemerin [112,113,114], it is mainly considered a pro-inflammatory cytokine/adipokine. Apart from being found in different inflammatory fluids [74,115,116,117,118], many immune cells express CMKLR1, including dendritic cells, macrophages, monocytes, and natural killer cells [86,119,120,121], which usually infiltrate adipose tissue in obesity [8] or the heart in pathological states [122]. Moreover, chemerin seems to be able to induce macrophage adhesion to extracellular matrix proteins and adhesion molecules, stimulating the recruitment and retention of macrophages at sites of inflammation [123]. Circulating levels of chemerin are also correlated with other pro-inflammatory markers such as TNFα, IL-6, and CRP [118,124,125,126,127] and with metabolic parameters such as an unfavourable lipid profile or increased glycated haemoglobin (HbA1c) [90]. In inflammatory diseases such as systemic sclerosis [126], CAD [127], ulcerative colitis, and Crohn’s disease [128], circulating chemerin levels are increased compared to controls. 

Apart from the indirect effects of chemerin on the cardiovascular pathophysiology due to its actions on the adipose tissue, insulin signalling, and inflammation at a systemic level, chemerin also exerts direct effects on different components of the cardiovascular system. Regarding the adipose depots that surround cardiac tissues, it has been described that chemerin expression in EAT correlates with the amount of EAT and is higher in patients with CAD [91,92]. Accordingly, it was reported that in periaortic abdominal and pericoronary adipose tissue from blood vessels with atherosclerotic lesions, chemerin expression correlates with the severity of atherosclerosis and is also expressed in foam cells and vascular smooth muscle cells (VSMCs) [83]. In patients with CAD, circulating levels of chemerin also correlate with the severity of CAD [129,130,131,132]. Some studies carried out in HUVECs have shown that chemerin participates in endothelial inflammation by inducing nuclear factor-κB (NF-κB), which is a key player in vascular inflammation, atherosclerosis development, and its pathological complications in atherothrombotic diseases [133,134] and the monocyte–endothelial adhesion [135]. In human VSMCs, chemerin has pro-apoptotic, pro-inflammatory, and proliferative effects that are mediated by nicotinamide adenine dinucleotide phosphate oxidase (Nox) activation and redox-sensitive mitogen-activated protein kinases signalling [136]. In glomerular endothelial cells, chemerin treatment increases the secretion of the pro-inflammatory cytokines TNFα, IL-6, IL-8, and TGF-β1, and exposure to high concentrations of glucose increases the mRNA levels of both chemerin and CMKLR1 in these cells [137]. However, an anti-inflammatory effect of chemerin in endothelial cells by the inhibition of NF-κB, the TNF-α-induced adhesion of monocytes, and the oxidised low-density lipoprotein (oxLDL)-induced macrophage foam cell formation has also been described, as was a reduction in the presence of pro-inflammatory markers [86,113]. Moreover, a 4-week infusion of chemerin-9, an agonist of CMKLR1, induces a decrease in aortic atherosclerotic lesions in apolipoprotein E-deficient (ApoE -/-) mice [86], while in another study using the same animal model, the contrary effect was reported, showing that chemerin protein levels correlated with inflammatory markers and plaque formation [138]. In the same line, although the majority of studies suggest that chemerin circulating levels are associated with the presence and severity of atherosclerosis [127,129,130,132,139,140,141,142], there are also contradictory results [124,143]. 

In the heart, chemerin has been shown to induce insulin resistance and apoptosis by decreasing AKT phosphorylation in neonatal rat cardiomyocytes [97,144], where chemerin, CMKLR1 mRNA, protein levels, and chemerin secretion are increased by TNFα and decreased by insulin [144]. Some studies have suggested a role of chemerin in cardiac contractility. In rat-perfused hearts, chemerin exerts a negative inotropic effect through the increase of endothelial nitric oxide synthase (eNOS) gene expression and cyclic guanosine monophosphate (cGMP) levels and the decrease of sarcolemmal L-type Ca2^+^ channel (CaV1.2) expression [145]. On the other hand, circulating chemerin levels are increased in patients with atrial fibrillation and atrial remodelling [127,140], a process where inflammation plays a key role due to the infiltration of immune cells and proteins that mediate the inflammatory response in the cardiac tissue, affecting calcium homeostasis and connexins and thus atrial electrophysiology [140]. 

In this line, several studies have shown that chemerin is able to promote contractility in blood vessels, including the pulmonary artery, aorta, and superior mesenteric artery in rats and the human saphenous vein [146,147,148,149,150]. It has also been demonstrated that chemerin promotes the increase of blood pressure in rats and mice [149,151,152,153]. These results are consistent with the presence of increased circulating levels of chemerin in patients with hypertension [90,125,154,155], where chemerin correlates with inflammatory markers such as TNFα, CRP, and IL-6; metabolic syndrome; and adipose tissue accumulation [90,125,154]. Some authors have suggested that the different adipose tissue depots could be responsible for the increased chemerin production and its effects on the blood vessels, proposing chemerin as a link between adipocytes and vasculature [136,148,153,156].

### 3.3. Resistin

Resistin was initially described by Steppan et al. in 2001 as a circulating protein expressed and secreted by white adipocytes that induces insulin resistance in rodents [157,158]. Its circulating levels in animal models of obesity and diabetes are increased, suggesting a deregulation of resistin production in these conditions, and both in vivo and in vitro studies have shown that resistin exerts an inhibitory effect on glucose uptake in murine adipocytes and muscle cells [158,159,160].

In humans, however, resistin is mainly expressed in peripheral blood mononuclear cells (PBMCs) (lymphocytes and monocytes), macrophages, and bone marrow cells but is also produced in smaller amounts in other cells/tissues, with its expression in the adipose tissue being due to non-adipocyte cells [161]. Resistin’s main role in humans seems to be pro-inflammatory. In human PBMCs, resistin increases the expression of the pro-inflammatory cytokines TNFα, IL-1β, and IL-6 in a mechanism dependent on NF-κB signalling [162]. In the same line, in human cultured macrophages, resistin expression is increased by lipopolysaccharide (LPS) and blocked by immunoneutralization of TNFα, IL-6, and IL-1β and by anti-inflammatory insulin-sensitizing drugs targeting NF-κB, indicating that for resistin production in these cells, it is necessary to induce a cascade of pro-inflammatory cytokines [163]. 

As mentioned above, resistin expression in humans is abundant in monocytes and macrophages, two cell types that play a crucial role in the development of atherosclerosis [164]. Accordingly, resistin levels were found to be increased in human atherosclerotic vessels and in atherosclerotic aneurysms due to its production by macrophages [165,166]. In EAT from patients with acute coronary syndrome, resistin expression and secretion is increased and associated with endothelial cell permeability [167]. In vascular endothelial cells, resistin induces the expression of adhesion molecules such as vascular cell adhesion molecule 1 (VCAM-1) and intercellular adhesion molecule 1 (ICAM-1), and inflammatory markers like monocyte chemoattractant protein-1 (MCP-1), long pentraxin 3, TNFα, IL-6, and IL-1β, as well as insulin, signalling impairment through the promotion of ROS production and endoplasmic reticulum stress [168,169,170]. In this line, in human coronary artery endothelial cells, it was shown that resistin also induces the production of ROS, which eventually induces mitochondrial dysfunction and an imbalance in cellular redox enzymes [165]. All of these observations suggest that resistin could have an important role in the pathogenesis of atherosclerosis by regulating the local inflammatory response in the blood vessels. 

Kawasaki disease (KD) is a syndrome characterised by acute febrile illness and systemic vasculitis of unknown aetiology that mainly affects young children, causing coronary artery aneurysms that, when untreated, can lead to ischaemic heart disease and myocardial infarction [171]. In KD children, circulating resistin levels are higher in those with coronary artery aneurysm compared with controls and KD without coronary artery aneurysm, and positively correlated with CRP. This indicates a role of resistin in the development of coronary artery aneurysms in this disease [172], an effect that seems to be mediated by inflammatory infiltration in the blood vessel and by the stimulation of the production of pro-inflammatory cytokines like TNFα and IL-1β in the coronary artery’s endothelial cells [173]. 

Recently, resistin has been proposed as a biomarker for the prediction of atrial arrhythmia recurrence after catheter ablation. Increased levels of circulating resistin in these patients are associated with poor left atrial substrate, high epicardial fat volume, and elevated circulating TNFα [174].

### 3.4. Oncostatin M

Oncostatin M (OSM) was discovered in 1986 as a tumour-inhibitory factor [175]. It is a pro-inflammatory cytokine that belongs to the gp130 family of cytokines, which also includes IL-6, IL-11, and leukaemia-inhibitory factor (LIF) [176]. It shows a pleiotropic effect, playing an important role in inflammation, cardiovascular and metabolic diseases, haematopoiesis, tissue remodelling, and cell growth and development [177,178,179].

OSM is produced by macrophages, monocytes, T cells, and dendritic cells. It is able to bind to two receptor complexes: heterodimers of gp130 with either the OSM receptor (OSMR) or the LIF receptor (LIFR) [180,181]. The binding of OSM to these receptors activates the janus kinase (JAK)/signal transducer and activator of transcription proteins (STAT), mitogen-activated protein kinase (MAPK)/extracellular signal-regulated kinase (ERK), and phosphoinositide 3-kinase (PI3K)/AKT serine/threonine kinase 1 (AKT), signalling pathways involved in the regulation of the inflammatory response and glucose modulation [182,183].

In obese mice, increased levels of macrophages in adipose tissue release this adipokine, which inhibits insulin-activated glucose transport to the tissues, inducing insulin resistance [181,184]. In humans, a relationship between insulin resistance indices and OSM plasma levels was also found [176].

OSM can also increase the effects of other inflammatory mediators [185]. For example, Rychli et al. demonstrated that this cytokine increases angiotensin 2 levels, thus inducing an inflammatory process, both in the human heart and in endothelial tissue [186].

Several studies have demonstrated the implication of OSM in cardiovascular diseases; in fact, its receptor is expressed in cardiomyocytes, which supports these findings. This adipokine is expressed in cardiac tissue from patients undergoing valve surgery, tissues from patients with end-stage heart failure and in aortic samples showing inflammation, but not in healthy hearts [186,187,188].

OSM plays a fundamental role in myocardial infarction by stimulating Reg3β, a crucial regulator of the sustained recruitment of macrophages that allow the elimination of damaged cardiomyocytes. However, the sustained activation of inflammatory pathways due to the action of the OMS promotes the progression of heart failure [180,188,189]. Setiadi et al. pointed out the possible relationship of OSM with thrombosis, highlighting that the endothelial signalling of OSM released by neutrophils increases P-selectin-dependent inflammation, increasing the recruitment of neutrophils and monocytes during the early stages of inflammation and thrombosis [190].

Furthermore, the presence of OSM has been detected both in human atherosclerotic lesions and in the mouse ApoE^−/−^ model of atherosclerosis [191]. It has been shown that the synergistic action of OSM with LPS activates MCP-1, IL-6, and vascular endothelial growth factor (VEGF) in aortic smooth muscle cells, increasing the levels of cytokines and growth factors present in atherosclerotic plaques, contributing to angiogenesis and plate destabilization [177,183,185,192,193]. 

### 3.5. Adiponectin

Adiponectin is a 30 kDa adipokine that exerts its actions by binding to the adiponectin receptors AdipoR1 and AdipoR2 [194,195]. Adiponectin induces energy expenditure and fatty acid oxidation, as well as the inhibition of food intake in obese rats, when administered chronically [196,197]. 

In the heart, AdipoR1 overexpression reduces hypertrophy and lipid accumulation in diet-induced obese mice through a mechanism that involves oxidative stress and autophagy reduction [198]. In isolated working hearts from T2DM mice lacking AdipoR1, an impairment of the myocardial mitochondrial function and coupling is observed [199], an effect that is preserved in healthy mice lacking adiponectin [200], which suggests that adiponectin signalling could have differential roles in the regulation of cardiac biology in pathological and non-pathological conditions. 

Contrary to leptin, circulating adiponectin levels are decreased in obesity and are inversely correlated with the body mass index (BMI), glycaemia, and circulating insulin levels, as well as with the risk of developing T2DM, obesity, and CVDs [201,202]. At a cardiovascular level, hypoadiponectinemia is related to impaired vasoreactivity and endothelial dysfunction, hypertension, coronary heart disease, or valvular inflammation [203,204,205,206,207,208,209].

Adiponectin is considered an anti-inflammatory adipokine. It reduces the expression of pro-inflammatory markers and oxidative stress, improving insulin resistance and preventing atherosclerosis [210]. Serum adiponectin is negatively correlated with pro-inflammatory markers such as CRP, IL-6, and TNFα, and it has an anti-inflammatory effect on the endothelium by suppressing the expression of adhesion molecules and by reducing the production of pro-inflammatory cytokines through the inhibition of NF-κβ [211,212,213,214,215]. Accordingly, it was shown that adiponectin and TNFα are reciprocally regulated in micro- and macrocirculation through NF-κβ signalling in T2DM mice. This means that adiponectin suppresses TNFα expression and TNFα blockage increases adiponectin expression in coronary arterioles and aorta, regulating endothelial dysfunction and suggesting a role of adiponectin in the prevention of vascular damage in T2DM [216]. Moreover, different studies have shown that hypoadiponectinemia causes endothelium dysfunction in mice and men [203,217,218], while adiponectin treatment counteracts endothelial dysfunction in obese rats by increasing NO production and eNOS phosphorylation [219]. Particularly in diabetic vascular endothelial dysfunction, hypoadiponectinemia is associated with NLRP3 inflammasome activation [204], which is implicated in the innate immune system response through the activation of caspase-1 and the secretion of pro-inflammatory cytokines IL-1β/IL-18 after microbial infection and cellular damage. In addition, when activated in aberrant circumstances, it is related to diabetes and atherosclerosis development [220]. In EAT from CAD patients, adiponectin levels are reduced at the same time that the production of IL-6, TNFα, and TLR-4 is increased, while adiponectin treatment has been proved to prevent atherosclerosis by reducing TNFα production in macrophages and ROS production by endothelial cells, as well as by increasing endothelial cell migration and vascularization [196,212,221]. 

Adiponectin is expressed and secreted by human and murine cardiomyocytes, where it enhances glucose and fatty acid uptake, suggesting a role of adiponectin in regulating cardiac metabolism and function [222]. In hearts from adiponectin-knockout mice, there is an increased expression of endoplasmic reticulum stress and inflammatory genes such as TNFα and MCP-1, an effect that is reverted by adiponectin treatment of H9C2 cardiomyocytes with induced endoplasmic reticulum stress and in HUVECs in sepsis [223,224]. In the same line, several studies have shown that adiponectin also protects the heart from I/R injury through the inhibition of endoplasmic reticulum stress [225,226,227] and that TNFα antagonism improves myocardial I/R injury by upregulating adiponectin expression in mice [228].

### 3.6. Nesfatin-1

Nesfatin-1 is an 82-amino acid peptide identified in 2006 in rats as a new hypothalamic molecule implicated in the regulation of food intake that promotes anorexia through a leptin-independent melanocortin signalling system. It is proteolytically cleaved from its precursor protein, nucleobindin-2 (NUCB2), which, depending on its differential processing, can lead to three polypeptides: nesfatin-1, nesfatin-2, and nesfatin-3, with the anorexigenic effect described only for nesfatin-1 [229]. Apart from the hypothalamus, nesfatin-1 was further shown to be produced by many peripheral tissues in humans, mice, and rats and to exert a wide range of biological effects through endocrine/autocrine/paracrine signalling in central and peripheral organs, although the nesfatin-1 receptor has not been identified so far [230]. Besides its anorexigenic function, nesfatin-1 regulates glucose homeostasis, gastric emptying and motility, the reproductive function, and anxiety and stress responses, among others [230]. 

Nesfatin-1 is expressed and secreted by human and murine adipose tissue, and its adipose, hypothalamic, and circulating levels are decreased by starvation and increased after refeeding or high-fat diet [229,231]. In severely obese patients, induced weight loss due to biliopancreatic diversion with duodenal switch also decreases circulating nesfatin-1 levels, which correlate with parameters of metabolic health after 12 months of the intervention, including improvements in weight, fat mass, fasting insulin levels and insulin resistance, cholesterol levels, and CRP circulating levels [232]. On the other hand, nesfatin-1 is also produced in the pancreas, where it regulates insulin secretion [233]. Taken together, these data suggest a physiological role of nesfatin-1 in energy metabolism regulation independently of its hypothalamic anorexigenic effects. However, there are contradictory results regarding circulating nesfatin-1 levels associated with the body mass index and T2DM [230]. 

Recently, some works have pointed to a possible inflammatory role for nesfatin-1 in different scenarios, with contradictory results. In adipose tissue, nesfatin-1 expression and secretion by 3T3-L1 cells and by subcutaneous adipose tissue explants are upregulated by both pro- and anti-inflammatory factors, indicating a possible role of nesfatin-1 in inflammatory states associated with obesity [231]. In the rat brain, some nesfatin-1-expressing neurons are activated during an inflammatory stimulus (LPS), indicating that nesfatin-1 may participate in the onset of physiological and behavioural changes that occur during acute-phase reactions due to infection and inflammation [234]. In pathologies with a clear inflammatory basis, such as osteoarthritis, lung injury, traumatic brain, or subarachnoid haemorrhage, nesfatin-1 has been associated with a protective effect due to a decrease in the oxidative stress and the inflammatory response in animal models [235,236,237,238]. However, in patients with spontaneous subarachnoid haemorrhage, circulating nesfatin-1 levels are increased and associated with the presence, size, and number of aneurysms, suggesting that nesfatin-1 could be a player in the inflammation response that causes the rupture of the aneurysmal sac [239].

Nesfatin-1 is expressed in rat and human hearts. In the human heart, NUCB2 mRNA expression is higher in women than men and higher in women with CAD than in healthy women [240]. According to this observation, in a study carried out in men and women undergoing elective coronary angiography, circulating nesfatin-1 levels were increased in patients with CAD, correlated with the number of >50% stenotic coronary segments and associated with CAD independently of atherosclerotic risk factors, suggesting that high nesfatin-1 levels in patients with CAD may play a role in the development of coronary atherosclerosis [241]. However, in patients diagnosed with non-ST segment elevation myocardial infarction, circulating nesfatin-1 levels were found to be decreased compared to patients with normal coronary artery and negatively correlated with CAD severity [242,243], an observation also found in patients with AMI [244] and with ST-segment elevation myocardial infarction [245]. In the same line, it has been shown that after 3 months of a coronary artery bypass operation due to atherosclerotic coronary artery disease, circulating nesfatin-1 levels increase compared to its preoperative levels, which suggests a possible cardioprotective effect of nesfatin-1 on revascularization [246]. 

Although little is known about the mechanism by which nesfatin-1 affects MI and CAD progression, a couple of studies carried out in rats have proposed that nesfatin-1 could act as an anti-inflammatory mediator with cardioprotective effects in these pathologies. Isoproterenol-induced MI rats are characterised by increased myocardial expression of pro-inflammatory cytokines like IL-1β, IL-6, and TNFα, as well as an increased number of apoptotic and necrotic cells in the myocardium. Nesfatin-1 administration to these rats has been shown to exert a beneficial effect by decreasing the expression of pro-inflammatory markers and apoptotic and necrotic cells in the cardiac tissue through AKT and GSK3β signalling, suggesting that nesfatin-1 could have anti-apoptotic and anti-inflammatory properties in the heart after suffering an MI [247]. Accordingly, in a rat model of MI established via ligation of the left anterior descending coronary artery, nesfatin-1 infusion prior to reperfusion decreased inflammation, oxidative stress, autophagy, and apoptosis in the heart after 24 h reperfusion [248].

### 3.7. Relaxin

Relaxin is a 6 kDa polypeptide hormone in the insulin/relaxin superfamily that was first identified as a reproductive hormone implicated in the lengthening of pubic symphysis of the birth canal during delivery and in vasoregulation during pregnancy, among other reproductive functions [249]. Although there is not much information regarding relaxin production by the adipose tissue, according to the database The Human Protein Atlas, relaxin mRNA expression is found in small amounts in adipose tissue (https://www.proteinatlas.org/ENSG00000107014-RLN2/tissue). Some studies carried out in the 1980s suggest that relaxin can affect adipocyte biology by increasing insulin binding in adipocytes derived from pregnant women and insulin binding and signalling in rat adipocytes [250,251] and by increasing lipid deposition in parametrial adipose cells from mice [252]. Taken together, these data suggest that the adipose tissue could be a producer of and a responder to relaxin under certain conditions, but more studies are needed. 

Relaxin is considered a pleiotropic hormone that exerts numerous favourable cardiovascular effects, suggesting its potential use for cardiovascular clinical purposes due to its anti-fibrotic, wound-healing, vasodilator, angiogenic, anti-hypertrophic, anti-apoptotic, anti-oxidant, and anti-inflammatory properties [253]. Relaxin highlights its anti-inflammatory role principally in conditions of MI following I/R injury. Relaxin inhibits cardiac mast cell activation and exocytosis degranulation in conjunction with a decrease in pro-inflammatory cytokines, including histamine, serotonin, and leukotrienes, and suppresses the activation and aggregation of platelets through the endogenous production of NO, the attenuation of intracellular calcium overload, and the decrease in malonyldialdehyde production in states of myocardial injury after I/R [254,255,256,257,258]. Moreover, in the myocardium, relaxin can reduce rat coronary endothelial cell adhesiveness to neutrophils by a decrease in VCAM-1 and P-selectin expression, as well as by neutrophils activation and migration through NO-dependent mechanisms and a decrease in the activity of myeloperoxidase (a marker of neutrophil accumulation) [256,259,260]. Relaxin blunts the NLRP3 inflammasome (which induces the synthesis of IL-1β and IL-18 in leukocytes and cardiomyocyte pyroptosis and apoptosis as well as increases the risk of MI) via the attenuation of caspase-1 activity through an eNOS-dependent mechanism in conditions of I/R injury [261,262]. Additionally, relaxin reduced the expression of the pro-inflammatory cytokines IL-6, IL-1β, TNFα, and MCP-1 and decreased macrophage infiltration in mice hearts with MI [263,264,265]. This deregulation of cytokine production, in combination with a decline in leukocyte density and an attenuation of endothelial leakage mediated by relaxin, helps to blunt the microvascular damage after events of cardiac IR [266]. It is important to note that an exacerbated inflammatory response during MI complications could induce a pro-fibrotic cardiac state with an activation of myofibroblasts and the consequent increase in the risk of arrhythmias and atrial fibrillation; conditions where relaxin is considered a promising therapeutic strategy mainly due to its anti-fibrotic, anti-inflammatory, and antioxidant roles [267,268,269]. Relaxin could also suppress the inflammatory and immune signalling pathways that are stimulated in aged rat hearts (principal risk factor of heart failure and atrial fibrillation). These pathways are decreased by relaxin in a gender-dependent manner and include tissue macrophage infiltration, activation of complement components (C3a and C4a), NF-κB signalling (which regulates several inflammatory genes), calcium-induced T lymphocyte apoptosis pathway, iCOS-iCOSL signalling in T-helper cells (which participates in activation and migration of lymphocytes and secretion of pro- and anti-inflammatory cytokines), nuclear factor of activated T-cells (NFAT) regulation of the immune response, TH1 signalling (which induces a pro-inflammatory response), and maturation of dendritic cells, as well as IL-6, interferon γ (IFNγ), and TLR4 inhibition and a decrease in gene expression of major histocompatibility complex [270]. The early vascular inflammation is mitigated by relaxin in HUVECs, human aortic smooth muscle cells, and THP-1 monocytes through a decrease in monocyte adhesion, an inhibition of the TNFα-induced expression of endothelial adhesion molecules (VCAM-1 and platelet endothelial cell adhesion molecule (PECAM)), and a decrease in MCP-1 and its receptor CCR-2 [271]. Curiously, relaxin is able to interact and signal through the glucocorticoid receptor (GR) in order to mitigate pro-inflammatory cytokine secretion (IL-1, IL-6 and TNFα) in human THP-1 monocytes in a relaxin family peptide receptor 1 (RXFP1)-independent manner [272]. All these effects have drawn attention to relaxin’s roles in mitigating the inflammatory response, reducing tissue injury, and fluid overload, which alleviate cardiac congestion and long-term complications of cardiovascular pathologies by exerting a positive impact on cardiac, vascular, hepatic, and renal dysfunction [270,273,274,275].

### 3.8. Omentin

Omentin, also known as intelectin-1, lactoferrin receptor, or endothelial lectin, is a secreted protein first described in intestinal cells in mice and humans in 1998 and 2001, respectively [276,277], and in human endothelial cells in 2001 [278]. Subsequently, it was identified in an omental fat cDNA library in 2005 in humans [279]. Later on, it was shown that omentin is mainly produced and secreted by VAT, while its expression in SAT is markedly low or even undetectable in some cases [280,281,282], and is also produced in EAT [283,284,285] and in PVAT [281,286], with WAT considered its main source of production. 

In obesity, omentin circulating and expression levels in VAT are decreased, correlating positively with plasma adiponectin and high-density lipoprotein (HDL) levels and negatively with the waist circumference, BMI, leptin and fasting insulin levels [287], and it is considered a marker of leanness. Omentin has been shown to exert a positive effect on insulin signalling and inflammation. In EAT from diabetic and non-diabetic patients, omentin treatment improves the insulin-induced glucose uptake under normo- and hyperglycemic conditions, and it enhances the adipogenesis-induced adiponectin expression and reduces TNFα expression in mature adipocytes, while it increases TNFα expression in stromal cells [288]. Recently, it has been shown that omentin overexpression restores glucose and insulin intolerance and improves insulin sensitivity in obese mice. At the same time, omentin decreases the production of pro-inflammatory cytokines like TNFα, IL-6, and IL-1β and increases the production of anti-inflammatory cytokines like adiponectin and IL-10, both in the adipose tissue from obese mice and in RAW 264.7 macrophages co-cultured with LPS, via inhibition of the thioredoxin-interacting protein (TXNIP)/NLRP3 signalling pathway [289]. Similarly, in human U937 macrophages treated with LPS, omentin was reported to exert a protective effect against oxidative stress; mitochondrial dysfunction; the expression and secretion of pro-inflammatory cytokines like IL-6, IL-18, and MCP-1; the expression of cyclooxygenase-2 (COX2); the secretion of prostaglandin E2 (PGE2), through the inhibition of the TLR-4/myeloid differentiation factor 88 (MyD88)/NF-κB signalling pathway [290].

At a cardiovascular level, reduced circulating omentin levels are associated with poor cardiac outcome in patients with heart failure and with the presence of hypertrophic and dilated cardiomyopathy, and they are considered an independent risk factor for the development of peripheral arterial disease and AMI [291,292,293,294,295,296,297]. Particularly in AMI patients, serum omentin levels significantly increase after 6 months of follow-up, being inversely correlated with CRP and IL-18, suggesting that the increase of omentin could mediate the reduction of inflammation 6 months after the AMI [298]. In patients with atrial fibrillation and cardiac valve disease, omentin expression was described to be downregulated in EAT and in right atrial appendages [299].

Omentin has been proposed as a protective molecule against endothelial dysfunction. In obese patients, the known decrease of circulating omentin levels is associated with endothelial dysfunction [300], while in patients with T2DM, increased circulating omentin levels are positively associated with the improvement of the endothelial function [301]. This effect was also observed in another study enrolling non-T2DM patients, in which increased circulating omentin levels were further associated with increased insulin sensitivity and with reduced BMI, systolic and diastolic blood pressure, and IL-6 and CRP-1 levels [302]. Accordingly, in isolated mouse aortas and mouse aortic endothelial cells, omentin was shown to protect against vascular-endothelial dysfunction induced by high glucose through the inhibition of endoplasmic reticulum and oxidative stress and by increasing NO production via activation of AMPK/peroxisome proliferator-activated receptor δ (PPARδ) pathway [303]. In HUVECs, omentin has been reported to inhibit TNFα-induced COX2 expression by the inhibition of C-Jun N-terminal kinase (JNK) signalling, exerting an inhibitory role on the inflammatory state of vascular endothelial cells [304]. It has also been reported to inhibit TNFα-induced expression of the adhesion molecules ICAM-1 and VCAM-1 [305] and to protect them against cell death caused by ROS [306]. In the same line, it was also reported in HUVECs that omentin can protect from free fatty acid-induced cell proliferation and migration and to reduce ICAM-1, MCP-1, NF-κB, Il-6, IL-1, and TNFα expression [299].

Regarding CAD, circulating omentin levels were shown to be decreased in patients with either CAD alone or its combination with T2DM compared to controls. After cardiac surgery, only patients without CAD or T2DM showed an increase in omentin circulating levels, suggesting that this increase could be a protective mechanism to help the myocardium overcome the surgery-induced inflammatory and stress responses in healthier patients [284]. In addition, omentin EAT expression was reported to be reduced in CAD patients compared to non-CAD patients, being lower in EAT areas surrounding coronary segments with stenosis than in those without stenosis [283]. However, another study showed that omentin expression in EAT from CAD patients was increased compared to controls, while its circulating levels were decreased, suggesting a possible local role of omentin in the development of CAD [285]. 

### 3.9. Meteorin-Like Hormone

Meteorin-like (Metrnl) is a novel small (~27kDa) secreted adipocytokine that has a beneficial effect on glucose homeostasis and that also functions as a novel immunoregulatory cytokine associated with anti-inflammatory effects [307,308,309]. Metrnl has been proposed to connect adaptive responses to the regulation of energy homeostasis and tissue inflammation and to have therapeutic potential for metabolic and inflammatory diseases [310]. Increased circulating levels of Metrnl activate energy expenditure, enhance glucose tolerance and induce the expression of genes associated with beige fat thermogenesis and anti-inflammatory actions [310,311]. On the contrary, it has been reported that serum levels of Mtrnl are decreased in patients with coronary artery disease, in which they negatively correlate with the levels of inflammatory cytokines [312,313]. In fact, low serum Metrnl is being considered as a possible alternative marker of endothelial dysfunction and atherosclerosis, independently of being a risk factor of T2DM [314].

Recently, it has been reported that Metrnl is able to alleviate the I/R injury in cultured cardiomyocytes by means of reducing endoplasmic reticulum stress, a process tightly linked to an anomalous production of inflammatory cytokines and subsequent cardiac cell apoptosis [122,315], and these findings have added interest to the study of the cardiovascular effects of this hormone for possible future therapeutic applications. 

### 3.10. Fibroblast Growth Factor 21

Fibroblast growth factor-21 (FGF-21) is a member of the fibroblast growth factor family expressed in multiple organs, such as metabolic organs like the WAT (including EAT and PVAT), liver and pancreas [316,317,318,319]. It is a secreted protein that acts mainly as an important endocrine metabolism regulator by inducing weight loss and controlling insulin signalling and glucose and lipid metabolism [316]. It was also described to have a well-characterised anti-inflammatory effect on different tissues/cells, including obese adipose tissue [320,321], liver [322], pancreas [323], lungs [324], heart [325], skeletal muscle [326], and macrophages [327,328].

Several studies have shown a protective role of FGF-21 against atherosclerosis via the regulation of different signalling pathways involved in inflammation, oxidative stress, cholesterol synthesis, and cell viability. In ApoE^−/−^ mice, FGF-21 treatment was described to mitigate atherosclerosis through the inhibition of NLRP3, the inhibition of factor-associated suicide (FAS) signalling, the reduction of cholesterol accumulation through the promotion of autophagy and by suppressing hepatic cholesterol synthesis, increasing adiponectin production by adipocytes, and attenuating endoplasmic-reticulum-stress-induced apoptosis [329,330,331,332,333]. In rats with atherosclerosis, FGF-21 decreases inflammation by increasing the signalling of nuclear factor erythroid 2-related factor 2 (Nrf2)-antioxidant response elements (ARE) and by reducing the expression of NF-κB [334,335]. In diabetic mice, FGF-21 ameliorates endothelial dysfunction by suppressing oxidative stress and enhancing endothelium-dependent vasorelaxation through the activation of calcium/calmodulin-dependent protein kinase kinase 2 (CaMKK2)/protein kinase AMP-activated catalytic subunit alpha (AMPKα) [336].

In EAT from T2DM patients with multivessel CAD, FGF-21 gene expression is reduced [317], and in patients undergoing cardiac surgery, FGF-21 expression in EAT increases after surgery, suggesting a role protecting from surgery-related inflammatory response [319]. However, several studies have shown that increased levels of circulating FGF-21 are associated with the presence of atherosclerosis [337,338,339,340,341], although there are contradictory results [342].

In the heart, FGF-21 prevents the cardiac damage observed in mice with diabetic cardiomyopathy by ameliorating lipotoxicity and oxidative stress [343]. In high-fat-fed rats, FGF-21 improves left ventricular function, as well as insulin signalling and inflammation [344]. Several independent studies have shown a protective role of FGF-21 on cardiac hypertrophy [345,346,347], and on ventricular arrhythmias in post-infarcted hearts [348] by reducing inflammation and/or oxidative stress.

## 4. Conclusions

Obesity, particularly adipose tissue dysfunction and ectopic fat accumulation, is closely related to the development of a systemic and local inflammatory state due to the imbalance in the production/release of adipokines and cytokines by the cells that conform to the adipose tissue. Pro-inflammatory adipose secretions released into the blood-stream or in adjacent tissues reach and affect the cardiovascular system either indirectly, by promoting risk factors for the development of cardiovascular diseases like insulin resistance, oxidative stress, inflammation, or atherosclerosis, or directly, affecting the biology of the heart and blood vessels. 

In recent years, numerous research studies have been focused on the implication of adipokines in the inflammatory response in different pathologies. Particularly at the cardiovascular level, certain adipokines with a clear pro-/anti-inflammatory effect seem to be good candidates to be therapeutically targeted in order to prevent or ameliorate CMDs. However, there still arise some contradictory results that oblige the scientific community to keep investigating adipokines to try to achieve a better knowledge about their function or to define a specific therapeutic intervention under certain conditions.

## Abbreviations

AKT	AKT serine/threonine kinase 1
AMI	Acute myocardial infarction
ApoE^−/−^	Apolipoprotein E double knockout
ARE	Antioxidant response elements
BMI	Body mass index
CAD	Coronary artery disease
CAP1	Adenylyl cyclase-associated protein 1
CaV1.2	L-type Ca2^+^ channel
CCRL2	C-C chemokine receptor-like 2
cGMP	Cyclic guanosine monophosphate
CMDs	Cardiometabolic diseases
CMKLR1	Chemokine-like receptor 1
COX2	Cyclooxygenase-2
CRP	C-reactive protein
CVDs	Cardiovascular diseases
DCN	Decorin
EAT	Epicardial adipose tissue
ERK	Extracellular signal-regulated kinase
FGF-21	Fibroblast growth factor-21
eNOS	Endothelial nitric oxide synthase
FAS	Factor-associated suicide
GPR1	G protein-coupled receptor 1
GR	Glucocorticoid receptor
HbA1c	Glycated haemoglobin
HDL	High-density lipoprotein
HUVECs	Human umbilical vein endothelial cells
I/R	Ischemia/reperfusion
ICAM-1	Intracellular molecular adhesion 1
IFNγ	Interferon γ
IGF-1R	Insulin growth factor-1 receptor
IL	Interleukin
JAK	Janus kinase
JNK	C-Jun N-terminal kinases
KD	Kawasaki disease
LepR	Leptin receptor
LIF	Leukaemia inhibitory factor
LIFR	Leukaemia inhibitory factor receptor
LPS	Lipopolysaccharide
MAPK	Mitogen-activated protein kinase
MCP-1	Monocyte chemoattractant protein-1
Metrnl	Meteorin-like
MI	Myocardial infarction
MyD88	Myeloid differentiation factor 88
NFAT	Nuclear factor of activated T-cells
NF-κβ	Nuclear factor
NLRP3	NOD-, LRR- and pyrin domain-containing protein 3
NO	Nitric oxide
Nox	Nicotinamide adenine dinucleotide phosphate oxidase
Nrf2	Nuclear factor erythroid 2-related factor 2
NUCB2	Nucleobindin 2
OSM	Oncostatin M
OSMR	Oncostatin M receptor
oxLDL	Oxidised low-density lipoprotein
PBMCs	Peripheral blood mononuclear cells
PECAM	Platelet endothelial cell adhesion molecule
PI3K	Phosphoinositide 3-kinase
PGE2	Prostaglandin E2
PPARδ	Peroxisome proliferator-activated receptor δ
PVAT	Perivascular adipose tissue
RARRES2	Retinoic acid receptor responder 2
ROR-1	Tyrosine kinase-like orphan receptor 1
ROS	Reactive oxygen species
RXFP1	Relaxin family peptide receptor 1
SAT	Subcutaneous adipose tissue
STAT	Signal transducer and activator of transcription protein
T2DM	Type 2 diabetes mellitus
TGF-β	Transforming growth factor β
TIG2	Tazarotene-induced gene 2
TLR-4	Toll-like receptor 4
TNFα	Tumour necrosis factor α
TXNIP	Thioredoxin-interacting protein
VAT	Visceral adipose tissue
VCAM-1	Vascular cell molecular adhesion 1
VEGF	Vascular endothelial growth factor
VSMCs	Vascular smooth muscle cells
WAT	White adipose tissue

## References

[1] World Health Organization Cardiovascular Diseases. https://www.who.int/health-topics/cardiovascular-diseases#tab=tab_1.

[2] Vegiopoulos A., Rohm M., Herzig S. (2017). Adipose tissue: Between the extremes. EMBO J..

[3] Dodson M.V., Du M., Wang S., Bergen W.G., Fernyhough-Culver M., Basu U., Poulos S.P., Hausman G.J. (2014). Adipose depots differ in cellularity, adipokines produced, gene expression, and cell systems. Adipocyte.

[4] Koliaki C., Liatis S., Kokkinos A. (2019). Obesity and cardiovascular disease: Revisiting an old relationship. Metabolism.

[5] Chait A., den Hartigh L.J. (2020). Adipose Tissue Distribution, Inflammation and Its Metabolic Consequences, Including Diabetes and Cardiovascular Disease. Front. Cardiovasc. Med..

[6] Longo M., Zatterale F., Naderi J., Parrillo L., Formisano P., Raciti G.A., Beguinot F., Miele C. (2019). Adipose Tissue Dysfunction as Determinant of Obesity-Associated Metabolic Complications. Int. J. Mol. Sci..

[7] Jung C.H., Lee W.J., Song K.-H. (2017). Metabolically healthy obesity: A friend or foe?. Kor. J. Intern. Med..

[8] Guzik T.J., Skiba D.S., Touyz R.M., Harrison D.G. (2017). The role of infiltrating immune cells in dysfunctional adipose tissue. Cardiovasc. Res..

[9] Fuster J.J., Ouchi N., Gokce N., Walsh K. (2016). Obesity-induced Changes in Adipose Tissue Microenvironment and Their Impact on Cardiovascular Disease. Circ. Res..

[10] Unamuno X., Gómez-Ambrosi J., Rodríguez A., Becerril S., Frühbeck G., Catalán V. (2018). Adipokine dysregulation and adipose tissue inflammation in human obesity. Eur. J. Clin. Invest..

[11] Bremer A.A., Jialal I. (2013). Adipose tissue dysfunction in nascent metabolic syndrome. J. Obes..

[12] Ferrara D., Montecucco F., Dallegri F., Carbone F. (2019). Impact of different ectopic fat depots on cardiovascular and metabolic diseases. J. Cell. Physiol..

[13] Rodríguez A., Becerril S., Hernández-Pardos A.W., Frühbeck G. (2020). Adipose tissue depot differences in adipokines and effects on skeletal and cardiac muscle. Curr. Opin. Pharmacol..

[14] Landecho M.F., Tuero C., Valentí V., Bilbao I., de la Higuera M., Frühbeck G. (2019). Relevance of Leptin and Other Adipokines in Obesity-Associated Cardiovascular Risk. Nutrients.

[15] Neeland I.J., Poirier P., Després J.-P. (2018). Cardiovascular and Metabolic Heterogeneity of Obesity. Circulation.

[16] McLaughlin T., Lamendola C., Liu A., Abbasi F. (2011). Preferential Fat Deposition in Subcutaneous Versus Visceral Depots Is Associated with Insulin Sensitivity. J. Clin. Endocrinol. Metab..

[17] Fain J.N., Madan A.K., Hiler M.L., Cheema P., Bahouth S.W. (2004). Comparison of the release of adipokines by adipose tissue, adipose tissue matrix, and adipocytes from visceral and subcutaneous abdominal adipose tissues of obese humans. Endocrinology.

[18] González N., Moreno-Villegas Z., González-Bris A., Egido J., Lorenzo Ó. (2017). Regulation of visceral and epicardial adipose tissue for preventing cardiovascular injuries associated to obesity and diabetes. Cardiovasc. Diabetol..

[19] Packer M. (2018). Epicardial Adipose Tissue May Mediate Deleterious Effects of Obesity and Inflammation on the Myocardium. J. Am. Coll. Cardiol..

[20] Zhou M., Wang H., Chen J., Zhao L. (2020). Epicardial adipose tissue and atrial fibrillation: Possible mechanisms, potential therapies, and future directions. Pacing Clin. Electrophysiol..

[21] Le Jemtel T.H., Samson R., Ayinapudi K., Singh T., Oparil S. (2019). Epicardial Adipose Tissue and Cardiovascular Disease. Curr. Hypertens. Rep..

[22] Patel V.B., Shah S., Verma S., Oudit G.Y. (2017). Epicardial adipose tissue as a metabolic transducer: Role in heart failure and coronary artery disease. Heart Fail. Rev..

[23] Wang M., Zhao L., Liang H., Zhang C., Guan L., Li M. (2019). A new measurement site for echocardiographic epicardial adipose tissue thickness and its value in predicting metabolic syndrome. Adv. Clin. Exp. Med..

[24] Keresztesi A.A., Asofie G., Simion M.A., Jung H. (2018). Correlation between epicardial adipose tissue thickness and the degree of coronary artery atherosclerosis. Turk. J. Med. Sci..

[25] Villasante Fricke A.C., Iacobellis G. (2019). Epicardial Adipose Tissue: Clinical Biomarker of Cardio-Metabolic Risk. Int. J. Mol. Sci..

[26] Erkan A.F., Tanindi A., Kocaman S.A., Ugurlu M., Tore H.F. (2016). Epicardial Adipose Tissue Thickness Is an Independent Predictor of Critical and Complex Coronary Artery Disease by Gensini and Syntax Scores. Texas Hear. Inst. J..

[27] Lima-Martínez M.M., Colmenares L., Campanelli Y., Paoli M., Rodney M., Santos R.D., Iacobellis G. (2019). Epicardial adipose tissue thickness and type 2 diabetes risk according to the FINDRISC modified for Latin America. Clín. Investig. Arterioscler..

[28] Hu H., Garcia-Barrio M., Jiang Z., Chen Y.E., Chang L. (2020). Roles of Perivascular Adipose Tissue in Hypertension and Atherosclerosis. Antioxid. Redox Signal..

[29] Quesada I., Cejas J., García R., Cannizzo B., Redondo A., Castro C. (2018). Vascular dysfunction elicited by a cross talk between periaortic adipose tissue and the vascular wall is reversed by pioglitazone. Cardiovasc. Ther..

[30] Qi X.-Y., Qu S.-L., Xiong W.-H., Rom O., Chang L., Jiang Z.-S. (2018). Perivascular adipose tissue (PVAT) in atherosclerosis: A double-edged sword. Cardiovasc. Diabetol..

[31] Spiroglou S.G., Kostopoulos C.G., Varakis J.N., Papadaki H.H. (2010). Adipokines in periaortic and epicardial adipose tissue: Differential expression and relation to atherosclerosis. J. Atheroscler. Thromb..

[32] Nosalski R., Guzik T.J. (2017). Perivascular adipose tissue inflammation in vascular disease. Br. J. Pharmacol..

[33] Anthony S.R., Guarnieri A.R., Gozdiff A., Helsley R.N., Phillip Owens A., Tranter M. (2019). Mechanisms linking adipose tissue inflammation to cardiac hypertrophy and fibrosis. Clin. Sci..

[34] Ahmadieh S., Kim H.W., Weintraub N.L. (2020). Potential role of perivascular adipose tissue in modulating atherosclerosis. Clin. Sci..

[35] Greenstein A.S., Khavandi K., Withers S.B., Sonoyama K., Clancy O., Jeziorska M., Laing I., Yates A.P., Pemberton P.W., Malik R.A. (2009). Local Inflammation and Hypoxia Abolish the Protective Anticontractile Properties of Perivascular Fat in Obese Patients. Circulation.

[36] DeVallance E., Branyan K.W., Lemaster K.C., Anderson R., Marshall K.L., Olfert I.M., Smith D.M., Kelley E.E., Bryner R.W., Frisbee J.C. (2019). Exercise training prevents the perivascular adipose tissue-induced aortic dysfunction with metabolic syndrome. Redox Biol..

[37] Han F., Hou N., Liu Y., Huang N., Pan R., Zhang X., Mao E., Sun X. (2019). Liraglutide improves vascular dysfunction by regulating a cAMP-independent PKA-AMPK pathway in perivascular adipose tissue in obese mice. Biomed. Pharmacother..

[38] Dong M., Ren J. (2014). What fans the fire: Insights into mechanisms of leptin in metabolic syndrome-associated heart diseases. Curr. Pharm. Des..

[39] Considine R.V., Sinha M.K., Heiman M.L., Kriauciunas A., Stephens T.W., Nyce M.R., Ohannesian J.P., Marco C.C., McKee L.J., Bauer T.L. (1996). Serum immunoreactive-leptin concentrations in normal-weight and obese humans. N. Engl. J. Med..

[40] Martin S.S., Qasim A., Reilly M.P. (2008). Leptin Resistance. J. Am. Coll. Cardiol..

[41] Katsiki N., Mikhailidis D.P., Banach M. (2018). Leptin, cardiovascular diseases and type 2 diabetes mellitus. Acta Pharmacol. Sin..

[42] Gruzdeva O., Borodkina D., Uchasova E., Dyleva Y., Barbarash O. (2019). Leptin resistance: Underlying mechanisms and diagnosis. Diabetes Metab. Syndr. Obes. Targets Ther..

[43] Lee M.-W., Lee M., Oh K.-J. (2019). Adipose Tissue-Derived Signatures for Obesity and Type 2 Diabetes: Adipokines, Batokines and MicroRNAs. J. Clin. Med..

[44] Gainsford T., Willson T.A., Metcalf D., Handman E., McFarlane C., Ng A., Nicola N.A., Alexander W.S., Hilton D.J. (1996). Leptin can induce proliferation, differentiation, and functional activation of hemopoietic cells. Proc. Natl. Acad. Sci. USA.

[45] Feijóo-Bandín S., Portolés M., Roselló-Lletí E., Rivera M., González-Juanatey J.R.R., Lago F. (2015). 20 years of leptin: Role of leptin in cardiomyocyte physiology and physiopathology. Life Sci..

[46] An H.S., Lee J.Y., Choi E.B., Jeong E.A., Shin H.J., Kim K.E., Park K.-A., Jin Z., Lee J.E., Koh J.S. (2020). Caloric restriction reverses left ventricular hypertrophy through the regulation of cardiac iron homeostasis in impaired leptin signaling mice. Sci. Rep..

[47] Ren J. (2004). Leptin and hyperleptinemia—From friend to foe for cardiovascular function. J. Endocrinol..

[48] Soderberg S., Ahren B., Jansson J.-H., Johnson O., Hallmans G., Asplund K., Olsson T. (1999). Leptin is associated with increased risk of myocardial infarction. J. Intern. Med..

[49] Kain D., Simon A.J., Greenberg A., Ben Zvi D., Gilburd B., Schneiderman J. (2018). Cardiac leptin overexpression in the context of acute MI and reperfusion potentiates myocardial remodeling and left ventricular dysfunction. PLoS ONE.

[50] Demarchi A., Mazzucchelli I., Somaschini A., Cornara S., Dusi V., Mirizzi A.M., Ruffinazzi M., Crimi G., Ferlini M., Gnecchi M. (2020). Leptin affects the inflammatory response after STEMI. Nutr. Metab. Cardiovasc. Dis..

[51] Ekmen N., Helvaci A., Gunaldi M., Sasani H., Yildirmak S.T. (2016). Leptin as an important link between obesity and cardiovascular risk factors in men with acute myocardial infarction. Ind. Heart J..

[52] Khafaji H.A.R.H., Bener A.B., Rizk N.M., Al Suwaidi J. (2012). Elevated serum leptin levels in patients with acute myocardial infarction; correlation with coronary angiographic and echocardiographic findings. BMC Res. Notes.

[53] Zhang T., Yang P., Li T., Gao J., Zhang Y. (2019). Leptin Expression in Human Epicardial Adipose Tissue Is Associated with Local Coronary Atherosclerosis. Med. Sci. Monit..

[54] Gruzdeva O., Uchasova E., Dyleva Y., Borodkina D., Akbasheva O., Antonova L., Matveeva V., Belik E., Ivanov S., Sotnikov A. (2019). Adipocytes Directly Affect Coronary Artery Disease Pathogenesis via Induction of Adipokine and Cytokine Imbalances. Front. Immunol..

[55] Drosos I., Chalikias G., Pavlaki M., Kareli D., Epitropou G., Bougioukas G., Mikroulis D., Konstantinou F., Giatromanolaki A., Ritis K. (2016). Differences between perivascular adipose tissue surrounding the heart and the internal mammary artery: Possible role for the leptin-inflammation-fibrosis-hypoxia axis. Clin. Res. Cardiol..

[56] Wang J., Hang T., Cheng X.-M., Li D.-M., Zhang Q.-G., Wang L.-J., Peng Y.-P., Gong J.-B. (2015). Associations of C1q/TNF-Related Protein-9 Levels in Serum and Epicardial Adipose Tissue with Coronary Atherosclerosis in Humans. Biomed Res. Int..

[57] Puurunen V.-P., Kiviniemi A., Lepojärvi S., Piira O.-P., Hedberg P., Junttila J., Ukkola O., Huikuri H. (2017). Leptin predicts short-term major adverse cardiac events in patients with coronary artery disease. Ann. Med..

[58] Puurunen V.P., Lepojärvi E.S., Piira O.P., Hedberg P., Junttila M.J., Ukkola O., Huikuri H.V. (2016). High plasma leptin levels are associated with impaired diastolic function in patients with coronary artery disease. Peptides.

[59] Farcaş A.D., Rusu A., Stoia M.A., Vida-Simiti L.A. (2018). Plasma leptin, but not resistin, TNF-α and adiponectin, is associated with echocardiographic parameters of cardiac remodeling in patients with coronary artery disease. Cytokine.

[60] Chen M.-C., Wang J.-H., Lee C.-J., Hsu B.-G. (2018). Association between hyperleptinemia and cardiovascular outcomes in patients with coronary artery disease. Ther. Clin. Risk Manag..

[61] Du Y., Yang S.-H., Li S., Zhao X., Zhang Y., Sun D., Zhu C.-G., Wu N.-Q., Guo Y.-L., Xu R.-X. (2018). Increased Serum Leptin Levels in New-Onset, Untreated Female Patients with Coronary Artery Disease and Positively Associated with Inflammatory Markers. Ann. Nutr. Metab..

[62] Bickel C., Schnabel R.B., Zeller T., Lackner K.J., Rupprecht H.J., Blankenberg S., Sinning C., Westermann D. (2017). Predictors of leptin concentration and association with cardiovascular risk in patients with coronary artery disease: Results from the Athero Gene study. Biomarkers.

[63] Knudson J.D., Dincer Ü.D., Zhang C., Swafford A.N., Koshida R., Picchi A., Focardi M., Dick G.M., Tune J.D. (2005). Leptin receptors are expressed in coronary arteries, and hyperleptinemia causes significant coronary endothelial dysfunction. Am. J. Physiol. Circ. Physiol..

[64] Liu B., Qiao J., Hu J., Fan M., Zhao Y., Su H., Wang Z., Yu Q., Ma Q., Li Y. (2020). Leptin promotes endothelial dysfunction in chronic kidney disease by modulating the MTA1-mediated WNT/β-catenin pathway. Mol. Cell. Biochem..

[65] Korda M., Kubant R., Patton S., Malinski T. (2008). Leptin-induced endothelial dysfunction in obesity. Am. J. Physiol. Circ. Physiol..

[66] De Rosa S., Cirillo P., Pacileo M., Di Palma V., Paglia A., Chiariello M. (2009). Leptin stimulated C-reactive protein production by human coronary artery endothelial cells. J. Vasc. Res..

[67] Singh P., Hoffmann M., Wolk R., Shamsuzzaman A.S.M., Somers V.K. (2007). Leptin induces C-reactive protein expression in vascular endothelial cells. Arterioscler. Thromb. Vasc. Biol..

[68] Bouloumie A., Marumo T., Lafontan M., Busse R. (1999). Leptin induces oxidative stress in human endothelial cells. FASEB J..

[69] Cirillo P., Angri V., De Rosa S., Calì G., Petrillo G., Maresca F., D’Ascoli G.-L., Maietta P., Brevetti L., Chiariello M. (2010). Pro-atherothrombotic effects of leptin in human coronary endothelial cells. Thromb. Haemost..

[70] Rourke J.L., Dranse H.J., Sinal C.J. (2013). Towards an integrative approach to understanding the role of chemerin in human health and disease. Obes. Rev..

[71] Ernst M.C., Sinal C.J. (2010). Chemerin: At the crossroads of inflammation and obesity. Trends Endocrinol. Metab..

[72] Mattern A., Zellmann T., Beck-Sickinger A.G. (2014). Processing, signaling, and physiological function of chemerin. IUBMB Life.

[73] Du X.-Y., Leung L.L.K. (2009). Proteolytic regulatory mechanism of chemerin bioactivity. Acta Biochim. Biophys. Sin..

[74] Wittamer V., Franssen J.-D., Vulcano M., Mirjolet J.-F., Le Poul E., Migeotte I., Brézillon S., Tyldesley R., Blanpain C.C., Detheux M. (2003). Specific Recruitment of Antigen-presenting Cells by Chemerin, a Novel Processed Ligand from Human Inflammatory Fluids. J. Exp. Med..

[75] Zabel B.A., Nakae S., Zúñiga L., Kim J.-Y., Ohyama T., Alt C., Pan J., Suto H., Soler D., Allen S.J. (2008). Mast cell-expressed orphan receptor CCRL2 binds chemerin and is required for optimal induction of IgE-mediated passive cutaneous anaphylaxis. J. Exp. Med..

[76] Monnier J., Lewén S., O’Hara E., Huang K., Tu H., Butcher E.C., Zabel B.A. (2012). Expression, regulation, and function of atypical chemerin receptor CCRL2 on endothelial cells. J. Immunol..

[77] Monnier J., Zabel B., Butcher E. (2010). Regulation of the Atypical Chemerin Receptor, CCRL2, on Activated Brain Endothelial Cells. Clin. Immunol..

[78] De Henau O., Degroot G.-N., Imbault V., Robert V., De Poorter C., Mcheik S., Galés C., Parmentier M., Springael J.-Y. (2016). Signaling Properties of Chemerin Receptors CMKLR1, GPR1 and CCRL2. PLoS ONE.

[79] Kennedy A.J., Davenport A.P. (2018). International Union of Basic and Clinical Pharmacology CIII: Chemerin Receptors CMKLR1 (Chemerin 1) and GPR1 (Chemerin 2) Nomenclature, Pharmacology, and Function. Pharmacol. Rev..

[80] Nagpal S., Patel S., Jacobe H., DiSepio D., Ghosn C., Malhotra M., Teng M., Duvic M., Chandraratna R.A. (1997). Tazarotene-induced gene 2 (TIG2), a novel retinoid-responsive gene in skin. J. Invest. Dermatol..

[81] Özcan E., Saygun N.I., Ilıkçı R., Karslıoğlu Y., Muşabak U., Yeşillik S. (2018). Evaluation of chemerin and its receptors, ChemR23 and CCRL2, in gingival tissues with healthy and periodontitis. Odontology.

[82] Chamberland J.P., Berman R.L., Aronis K.N., Mantzoros C.S. (2013). Chemerin is expressed mainly in pancreas and liver, is regulated by energy deprivation, and lacks day/night variation in humans. Eur. J. Endocrinol..

[83] Kostopoulos C.G., Spiroglou S.G., Varakis J.N., Apostolakis E., Papadaki H.H. (2014). Chemerin and CMKLR1 expression in human arteries and periadventitial fat: A possible role for local chemerin in atherosclerosis?. BMC Cardiovasc. Disord..

[84] Berg V., Sveinbjörnsson B., Bendiksen S., Brox J., Meknas K., Figenschau Y. (2010). Human articular chondrocytes express ChemR23 and chemerin; ChemR23 promotes inflammatory signalling upon binding the ligand chemerin(21-157). Arthritis Res. Ther..

[85] Bongrani A., Mellouk N., Rame C., Cornuau M., Guérif F., Froment P., Dupont J. (2019). Ovarian Expression of Adipokines in Polycystic Ovary Syndrome: A Role for Chemerin, Omentin, and Apelin in Follicular Growth Arrest and Ovulatory Dysfunction?. Int. J. Mol. Sci..

[86] Sato K., Yoshizawa H., Seki T., Shirai R., Yamashita T., Okano T., Shibata K., Wakamatsu M.J., Mori Y., Morita T. (2019). Chemerin-9, a potent agonist of chemerin receptor (ChemR23), prevents atherogenesis. Clin. Sci..

[87] Goralski K.B., McCarthy T.C., Hanniman E.A., Zabel B.A., Butcher E.C., Parlee S.D., Muruganandan S., Sinal C.J. (2007). Chemerin, a novel adipokine that regulates adipogenesis and adipocyte metabolism. J. Biol. Chem..

[88] Alfadda A.A., Sallam R.M., Chishti M.A., Moustafa A.S., Fatma S., Alomaim W.S., Al-Naami M.Y., Bassas A.F., Chrousos G.P., Jo H. (2012). Differential patterns of serum concentration and adipose tissue expression of chemerin in obesity: Adipose depot specificity and gender dimorphism. Mol. Cells.

[89] Martínez-García M.Á., Montes-Nieto R., Fernández-Durán E., Insenser M., Luque-Ramírez M., Escobar-Morreale H.F. (2013). Evidence for Masculinization of Adipokine Gene Expression in Visceral and Subcutaneous Adipose Tissue of Obese Women With Polycystic Ovary Syndrome (PCOS). J. Clin. Endocrinol. Metab..

[90] Zylla S., Pietzner M., Kühn J.-P., Völzke H., Dörr M., Nauck M., Friedrich N. (2017). Serum chemerin is associated with inflammatory and metabolic parameters-results of a population-based study. Obesity.

[91] Gao X., Mi S., Zhang F., Gong F., Lai Y., Gao F., Zhang X., Wang L., Tao H. (2011). Association of chemerin mRNA expression in human epicardial adipose tissue with coronary atherosclerosis. Cardiovasc. Diabetol..

[92] Wu Q., Chen Y., Chen S., Wu X., Nong W. (2019). Correlation between adiponectin, chemerin, vascular endothelial growth factor and epicardial fat volume in patients with coronary artery disease. Exp. Ther. Med..

[93] Goralski K.B., Sinal C.J. (2009). Elucidation of chemerin and chemokine-like receptor-1 function in adipocytes by adenoviral-mediated shRNA knockdown of gene expression. Methods Enzymol..

[94] Takahashi M., Takahashi Y., Takahashi K., Zolotaryov F.N., Hong K.S., Kitazawa R., Iida K., Okimura Y., Kaji H., Kitazawa S. (2008). Chemerin enhances insulin signaling and potentiates insulin-stimulated glucose uptake in 3T3-L1 adipocytes. FEBS Lett..

[95] Kralisch S., Weise S., Sommer G., Lipfert J., Lossner U., Bluher M., Stumvoll M., Fasshauer M. (2009). Interleukin-1ß induces the novel adipokine chemerin in adipocytes in vitro. Regul. Pept..

[96] Sell H., Laurencikiene J., Taube A., Eckardt K., Cramer A., Horrighs A., Arner P., Eckel J. (2009). Chemerin is a Novel Adipocyte-Derived Factor Inducing Insulin Resistance in Primary Human Skeletal Muscle Cells. Diabetes.

[97] Zhang R., Liu S., Guo B., Chang L., Li Y. (2014). Chemerin Induces Insulin Resistance in Rat Cardiomyocytes in Part through the ERK1/2 Signaling Pathway. Pharmacology.

[98] Ernst M.C., Issa M., Goralski K.B., Sinal C.J. (2010). Chemerin Exacerbates Glucose Intolerance in Mouse Models of Obesity and Diabetes. Endocrinology.

[99] Takahashi M., Okimura Y., Iguchi G., Nishizawa H., Yamamoto M., Suda K., Kitazawa R., Fujimoto W., Takahashi K., Zolotaryov F.N. (2011). Chemerin regulates β-cell function in mice. Sci. Rep..

[100] Ernst M.C., Haidl I.D., Zúñiga L.A., Dranse H.J., Rourke J.L., Zabel B.A., Butcher E.C., Sinal C.J. (2012). Disruption of the Chemokine-Like Receptor-1 (CMKLR1) Gene Is Associated with Reduced Adiposity and Glucose Intolerance. Endocrinology.

[101] El-Deeb T.S., Bakkar S.M., Eltoony L., Zakhary M.M., Kamel A.A., Nafee A.M., Hetta H.F. (2018). The adipokine Chemerin and Fetuin-A Serum Levels in Type 2 Diabetes Mellitus: Relation to Obesity and Inflammatory Markers. Egypt. J. Immunol..

[102] Ba H.-J., Xu L.-L., Qin Y.-Z., Chen H.-S. (2019). Serum Chemerin Levels Correlate with Determinants of Metabolic Syndrome in Obese Children and Adolescents. Clin. Med. Insights. Pediatr..

[103] Yang M., Zhou X., Xu J., Yang B., Yu J., Gong Q., Zhang X., Sun X., Zhang Q., Xia J. (2019). Association of serum chemerin and inflammatory factors with type 2 diabetes macroangiopathy and waist-to-stature ratio. Bosn. J. Basic Med. Sci..

[104] Skuratovskaia D., Zatolokin P., Vulf M., Mazunin I., Litvinova L. (2019). Interrelation of chemerin and TNF-α with mtDNA copy number in adipose tissues and blood cells in obese patients with and without type 2 diabetes. BMC Med. Genomics.

[105] Liu M., Lin X., Wang X. (2018). Decrease in serum chemerin through aerobic exercise plus dieting and its association with mitigation of cardio-metabolic risk in obese female adolescents. J. Pediatr. Endocrinol. Metab..

[106] Chakaroun R., Raschpichler M., Klöting N., Oberbach A., Flehmig G., Kern M., Schön M.R., Shang E., Lohmann T., Dreßler M. (2012). Effects of weight loss and exercise on chemerin serum concentrations and adipose tissue expression in human obesity. Metabolism.

[107] Sell H., Divoux A., Poitou C., Basdevant A., Bouillot J.-L., Bedossa P., Tordjman J., Eckel J., Clément K. (2010). Chemerin Correlates with Markers for Fatty Liver in Morbidly Obese Patients and Strongly Decreases after Weight Loss Induced by Bariatric Surgery. J. Clin. Endocrinol. Metab..

[108] Kolahdouzi S., Baghadam M., Kani-Golzar F.A., Saeidi A., Jabbour G., Ayadi A., De Sousa M., Zouita A., Abderrahmane A.B., Zouhal H. (2019). Progressive circuit resistance training improves inflammatory biomarkers and insulin resistance in obese men. Physiol. Behav..

[109] Catalán V., Gómez-Ambrosi J., Rodríguez A., Ramírez B., Rotellar F., Valentí V., Silva C., Gil M.J., Salvador J., Frühbeck G. (2013). Increased levels of chemerin and its receptor, chemokine-like receptor-1, in obesity are related to inflammation: Tumor necrosis factor-α stimulates mRNA levels of chemerin in visceral adipocytes from obese patients. Surg. Obes. Relat. Dis..

[110] Roman A.A., Parlee S.D., Sinal C.J. (2012). Chemerin: A potential endocrine link between obesity and type 2 diabetes. Endocrine.

[111] Ghosh A.R., Bhattacharya R., Bhattacharya S., Nargis T., Rahaman O., Duttagupta P., Raychaudhuri D., Liu C.S.C., Roy S., Ghosh P. (2016). Adipose Recruitment and Activation of Plasmacytoid Dendritic Cells Fuel Metaflammation. Diabetes.

[112] Cash J.L., Hart R., Russ A., Dixon J.P.C., Colledge W.H., Doran J., Hendrick A.G., Carlton M.B.L., Greaves D.R. (2008). Synthetic chemerin-derived peptides suppress inflammation through ChemR23. J. Exp. Med..

[113] Yamawaki H., Kameshima S., Usui T., Okada M., Hara Y. (2012). A novel adipocytokine, chemerin exerts anti-inflammatory roles in human vascular endothelial cells. Biochem. Biophys. Res. Commun..

[114] Laranjeira S., Regan-Komito D., Iqbal A.J., Greaves D.R., Payne S.J., Orlowski P. (2018). A model for the optimization of anti-inflammatory treatment with chemerin. Interface Focus.

[115] Valcamonica E., Chighizola C.B., Comi D., De Lucia O., Pisoni L., Murgo A., Salvi V., Sozzani S., Meroni P.L. (2014). Levels of chemerin and interleukin 8 in the synovial fluid of patients with inflammatory arthritides and osteoarthritis. Clin. Exp. Rheumatol..

[116] Patnaik K., Pradeep A.R., Nagpal K., Karvekar S., Singh P., Raju A. (2017). Human chemerin correlation in gingival crevicular fluid and tear fluid as markers of inflammation in chronic periodontitis and type-2 diabetes mellitus. J. Investig. Clin. Dent..

[117] Calvet J., Orellana C., Gratacós J., Berenguer-Llergo A., Caixàs A., Chillarón J.J., Pedro-Botet J., García-Manrique M., Navarro N., Larrosa M. (2016). Synovial fluid adipokines are associated with clinical severity in knee osteoarthritis: A cross-sectional study in female patients with joint effusion. Arthritis Res. Ther..

[118] Huang K., Du G., Li L., Liang H., Zhang B. (2012). Association of chemerin levels in synovial fluid with the severity of knee osteoarthritis. Biomarkers.

[119] Zabel B.A., Ohyama T., Zuniga L., Kim J.-Y., Johnston B., Allen S.J., Guido D.G., Handel T.M., Butcher E.C. (2006). Chemokine-like receptor 1 expression by macrophages in vivo: Regulation by TGF-β and TLR ligands. Exp. Hematol..

[120] Parolini S., Santoro A., Marcenaro E., Luini W., Massardi L., Facchetti F., Communi D., Parmentier M., Majorana A., Sironi M. (2007). The role of chemerin in the colocalization of NK and dendritic cell subsets into inflamed tissues. Blood.

[121] Zabel B.A., Silverio A.M., Butcher E.C. (2005). Chemokine-Like Receptor 1 Expression and Chemerin-Directed Chemotaxis Distinguish Plasmacytoid from Myeloid Dendritic Cells in Human Blood. J. Immunol..

[122] Nishida K., Otsu K. (2017). Inflammation and metabolic cardiomyopathy. Cardiovasc. Res..

[123] Hart R., Greaves D.R. (2010). Chemerin Contributes to Inflammation by Promoting Macrophage Adhesion to VCAM-1 and Fibronectin through Clustering of VLA-4 and VLA-5. J. Immunol..

[124] Lehrke M., Becker A., Greif M., Stark R., Laubender R.P., von Ziegler F., Lebherz C., Tittus J., Reiser M., Becker C. (2009). Chemerin is associated with markers of inflammation and components of the metabolic syndrome but does not predict coronary atherosclerosis. Eur. J. Endocrinol..

[125] Gu P., Jiang W., Lu B., Shi Z. (2014). Chemerin is associated with inflammatory markers and metabolic syndrome phenotypes in hypertension patients. Clin. Exp. Hypertens..

[126] Sawicka K., Michalska-Jakubus M., Potembska E., Kowal M., Pietrzak A., Krasowska D. (2019). Visfatin and chemerin levels correspond with inflammation and might reflect the bridge between metabolism, inflammation and fibrosis in patients with systemic sclerosis. Adv. Dermatol. Allergol..

[127] Kammerer A., Staab H., Herberg M., Kerner C., Klöting N., Aust G. (2018). Increased circulating chemerin in patients with advanced carotid stenosis. BMC Cardiovasc. Disord..

[128] Weigert J., Obermeier F., Neumeier M., Wanninger J., Filarsky M., Bauer S., Aslanidis C., Rogler G., Ott C., Schäffler A. (2010). Circulating levels of chemerin and adiponectin are higher in ulcerative colitis and chemerin is elevated in Crohn’s disease. Inflamm. Bowel Dis..

[129] Xiaotao L., Xiaoxia Z., Yue X., Liye W. (2012). Serum chemerin levels are associated with the presence and extent of coronary artery disease. Coron. Artery Dis..

[130] Dong B., Ji W., Zhang Y. (2011). Elevated Serum Chemerin Levels are Associated with the Presence of Coronary Artery Disease in Patients with Metabolic Syndrome. Intern. Med..

[131] Aksan G., İnci S., Nar G., Soylu K., Gedikli Ö., Yüksel S., Özdemir M., Nar R., Meriç M., Şahin M. (2014). Association of serum chemerin levels with the severity of coronary artery disease in patients with metabolic syndrome. Int. J. Clin. Exp. Med..

[132] Motawi T.M.K., Mahdy S.G., El-Sawalhi M.M., Ali E.N., El-Telbany R.F.A. (2018). Serum levels of chemerin, apelin, vaspin, and omentin-1 in obese type 2 diabetic Egyptian patients with coronary artery stenosis. Can. J. Physiol. Pharmacol..

[133] Mussbacher M., Salzmann M., Brostjan C., Hoesel B., Schoergenhofer C., Datler H., Hohensinner P., Basílio J., Petzelbauer P., Assinger A. (2019). Cell Type-Specific Roles of NF-κB Linking Inflammation and Thrombosis. Front. Immunol..

[134] Kojok K., El-Kadiry A.E.-H., Merhi Y. (2019). Role of NF-κB in Platelet Function. Int. J. Mol. Sci..

[135] Dimitriadis G.K., Kaur J., Adya R., Miras A.D., Mattu H.S., Hattersley J.G., Kaltsas G., Tan B.K., Randeva H.S. (2018). Chemerin induces endothelial cell inflammation: Activation of nuclear factor-kappa beta and monocyte-endothelial adhesion. Oncotarget.

[136] Neves K.B., Nguyen Dinh Cat A., Lopes R.A.M., Rios F.J., Anagnostopoulou A., Lobato N.S., de Oliveira A.M., Tostes R.C., Montezano A.C., Touyz R.M. (2015). Chemerin Regulates Crosstalk Between Adipocytes and Vascular Cells Through Nox. Hypertension.

[137] Shang J., Wang L., Zhang Y., Zhang S., Ning L., Zhao J., Cheng G., Liu D., Xiao J., Zhao Z. (2019). Chemerin/ChemR23 axis promotes inflammation of glomerular endothelial cells in diabetic nephropathy. J. Cell. Mol. Med..

[138] Liu H., Xiong W., Luo Y., Chen H., He Y., Cao Y., Dong S. (2019). Adipokine Chemerin Stimulates Progression of Atherosclerosis in ApoE −/− Mice. Biomed Res. Int..

[139] Er L., Hsu L.-A., Juang J.-M., Chiang F.-T., Teng M.-S., Tzeng I.-S., Wu S., Lin J.-F., Ko Y.-L. (2019). Circulating Chemerin Levels, but not the RARRES2 Polymorphisms, Predict the Long-Term Outcome of Angiographically Confirmed Coronary Artery Disease. Int. J. Mol. Sci..

[140] Zhang G., Xiao M., Zhang L., Zhao Y., Yang Q. (2017). Association of serum chemerin concentrations with the presence of atrial fibrillation. Ann. Clin. Biochem..

[141] Zhao D., Bi G., Feng J., Huang R., Chen X. (2015). Association of Serum Chemerin Levels with Acute Ischemic Stroke and Carotid Artery Atherosclerosis in a Chinese Population. Med. Sci. Monit..

[142] Gu P., Cheng M., Hui X., Lu B., Jiang W., Shi Z. (2015). Elevating circulation chemerin level is associated with endothelial dysfunction and early atherosclerotic changes in essential hypertensive patients. J. Hypertens..

[143] Aydin K., Canpolat U., Akin S., Dural M., Karakaya J., Aytemir K., Ozer N., Gurlek A. (2015). Chemerin is not associated with subclinical atherosclerosis markers in prediabetes and diabetes. Anatol. J. Cardiol..

[144] Rodríguez-Penas D., Feijóo-Bandín S., García-Rúa V., Mosquera-Leal A., Durán D., Varela A., Portolés M., Roselló-Lletí E., Rivera M., Diéguez C. (2015). The adipokine chemerin induces apoptosis in cardiomyocytes. Cell. Physiol. Biochem..

[145] Kutlay Ö., Kaygısız Z., Kaygısız B. (2018). The Effect of Chemerin on Cardiac Parameters and Gene Expressions in Isolated Perfused Rat Heart. Balkan Med. J..

[146] Hanthazi A., Jespers P., Vegh G., Degroot G.-N., Springael J.-Y., Lybaert P., Dewachter L., Mc Entee K. (2019). Chemerin influences endothelin- and serotonin-induced pulmonary artery vasoconstriction in rats. Life Sci..

[147] Ferland D.J., Darios E.S., Neubig R.R., Sjögren B., Truong N., Torres R., Dexheimer T.S., Thompson J.M., Watts S.W. (2017). Chemerin-induced arterial contraction is Gi- and calcium-dependent. Vascul. Pharmacol..

[148] Darios E.S., Winner B.M., Charvat T., Krasinksi A., Punna S., Watts S.W. (2016). The adipokine chemerin amplifies electrical field-stimulated contraction in the isolated rat superior mesenteric artery. Am. J. Physiol. Circ. Physiol..

[149] Kennedy A.J., Yang P., Read C., Kuc R.E., Yang L., Taylor E.J.A., Taylor C.W., Maguire J.J., Davenport A.P. (2016). Chemerin Elicits Potent Constrictor Actions via Chemokine-Like Receptor 1 (CMKLR1), not G-Protein-Coupled Receptor 1 (GPR1), in Human and Rat Vasculature. J. Am. Heart Assoc..

[150] Lobato N.S., Neves K.B., Filgueira F.P., Fortes Z.B., Carvalho M.H.C., Webb R.C., Oliveira A.M., Tostes R.C. (2012). The adipokine chemerin augments vascular reactivity to contractile stimuli via activation of the MEK-ERK1/2 pathway. Life Sci..

[151] Kunimoto H., Kazama K., Takai M., Oda M., Okada M., Yamawaki H. (2015). Chemerin promotes the proliferation and migration of vascular smooth muscle and increases mouse blood pressure. Am. J. Physiol. Circ. Physiol..

[152] Weng C., Shen Z., Li X., Jiang W., Peng L., Yuan H., Yang K., Wang J. (2017). Effects of chemerin/CMKLR1 in obesity-induced hypertension and potential mechanism. Am. J. Transl. Res..

[153] Ferland D.J., Flood E.D., Garver H., Yeh S.T., Riney S., Mullick A.E., Fink G.D., Watts S.W. (2019). Different blood pressure responses in hypertensive rats following chemerin mRNA inhibition in dietary high fat compared to dietary high-salt conditions. Physiol. Genomics.

[154] Yang M., Yang G., Dong J., Liu Y., Zong H., Liu H., Boden G., Li L. (2010). Elevated plasma levels of chemerin in newly diagnosed type 2 diabetes mellitus with hypertension. J. Investig. Med..

[155] Meric M., Soylu K., Avci B., Yuksel S., Gulel O., Yenercag M., Coksevim M., Uzun A. (2014). Evaluation of plasma chemerin levels in patients with non-dipper blood pressure patterns. Med. Sci. Monit..

[156] Watts S.W., Dorrance A.M., Penfold M.E., Rourke J.L., Sinal C.J., Seitz B., Sullivan T.J., Charvat T.T., Thompson J.M., Burnett R. (2013). Chemerin connects fat to arterial contraction. Arterioscler. Thromb. Vasc. Biol..

[157] Steppan C.M., Brown E.J., Wright C.M., Bhat S., Banerjee R.R., Dai C.Y., Enders G.H., Silberg D.G., Wen X., Wu G.D. (2001). A family of tissue-specific resistin-like molecules. Proc. Natl. Acad. Sci. USA.

[158] Steppan C.M., Bailey S.T., Bhat S., Brown E.J., Banerjee R.R., Wright C.M., Patel H.R., Ahima R.S., Lazar M.A. (2001). The hormone resistin links obesity to diabetes. Nature.

[159] Moon B., Kwan J.J.-M., Duddy N., Sweeney G., Begum N. (2003). Resistin inhibits glucose uptake in L6 cells independently of changes in insulin signaling and GLUT4 translocation. Am. J. Physiol. Metab..

[160] Rajala M.W., Obici S., Scherer P.E., Rossetti L. (2003). Adipose-derived resistin and gut-derived resistin-like molecule–β selectively impair insulin action on glucose production. J. Clin. Invest..

[161] Acquarone E., Monacelli F., Borghi R., Nencioni A., Odetti P. (2019). Resistin: A reappraisal. Mech. Ageing Dev..

[162] Bokarewa M., Nagaev I., Dahlberg L., Smith U., Tarkowski A. (2005). Resistin, an Adipokine with Potent Proinflammatory Properties. J. Immunol..

[163] Lehrke M., Reilly M.P., Millington S.C., Iqbal N., Rader D.J., Lazar M.A. (2004). An Inflammatory Cascade Leading to Hyperresistinemia in Humans. PLoS Med..

[164] Plutzky J. (2001). Inflammatory pathways in atherosclerosis and acute coronary syndromes. Am. J. Cardiol..

[165] Chen C., Jiang J., Lü J.-M., Chai H., Wang X., Lin P.H., Yao Q. (2010). Resistin decreases expression of endothelial nitric oxide synthase through oxidative stress in human coronary artery endothelial cells. Am. J. Physiol. Circ. Physiol..

[166] Jung H.S., Park K.H., Cho Y.M., Chung S.S., Cho H.J., Cho S.Y., Kim S.J., Kim S.Y., Lee H.K., Park K.S. (2006). Resistin is secreted from macrophages in atheromas and promotes atherosclerosis. Cardiovasc. Res..

[167] Langheim S., Dreas L., Veschini L., Maisano F., Foglieni C., Ferrarello S., Sinagra G., Zingone B., Alfieri O., Ferrero E. (2010). Increased expression and secretion of resistin in epicardial adipose tissue of patients with acute coronary syndrome. Am. J. Physiol. Circ. Physiol..

[168] Kawanami D., Maemura K., Takeda N., Harada T., Nojiri T., Imai Y., Manabe I., Utsunomiya K., Nagai R. (2004). Direct reciprocal effects of resistin and adiponectin on vascular endothelial cells: A new insight into adipocytokine–endothelial cell interactions. Biochem. Biophys. Res. Commun..

[169] Verma S., Li S.-H., Wang C.-H., Fedak P.W.M., Li R.-K., Weisel R.D., Mickle D.A.G. (2003). Resistin Promotes Endothelial Cell Activation. Circulation.

[170] Luo J., Huang L., Wang A., Liu Y., Cai R., Li W., Zhou M.-S. (2018). Resistin-Induced Endoplasmic Reticulum Stress Contributes to the Impairment of Insulin Signaling in Endothelium. Front. Pharmacol..

[171] Noval Rivas M., Arditi M. (2020). Kawasaki disease: Pathophysiology and insights from mouse models. Nat. Rev. Rheumatol..

[172] Liu R., He B., Gao F., Liu Q., Yi Q. (2012). Relationship between adipokines and coronary artery aneurysm in children with Kawasaki disease. Transl. Res..

[173] Gao F., Si F., Feng S., Yi Q., Liu R. (2016). Resistin Enhances Inflammatory Cytokine Production in Coronary Artery Tissues by Activating the NF- κ B Signaling. Biomed Res. Int..

[174] Chang T.-Y., Hsiao Y.-W., Guo S.-M., Chang S.-L., Lin Y.-J., Lo L.-W., Hu Y.-F., Chung F.-P., Chao T.-F., Liao J.-N. (2020). Resistin as a Biomarker for the Prediction of Left Atrial Substrate and Recurrence in Patients with Drug-Refractory Atrial Fibrillation Undergoing Catheter Ablation. Int. Heart J..

[175] Zarling J.M., Shoyab M., Marquardt H., Hanson M.B., Lioubin M.N., Todaro G.J. (1986). Oncostatin M: A growth regulator produced by differentiated histiocytic lymphoma cells. Proc. Natl. Acad. Sci. USA.

[176] Akarsu M., Hurşitoğlu M., Toprak Z., Yoldemir Ş.A., Altun Ö., Toprak I.D., Özcan M., Yürüyen G., Uğurlukişi B., Erdem M.G. (2019). Relationships among oncostatin M, insulin resistance, and chronic inflammation: A pilot study. Arch. Endocrinol. Metab..

[177] Demyanets S., Kaun C., Rychli K., Pfaffenberger S., Kastl S.P., Hohensinner P.J., Rega G., Katsaros K.M., Afonyushkin T., Bochkov V.N. (2011). Oncostatin M-enhanced vascular endothelial growth factor expression in human vascular smooth muscle cells involves PI3K-, p38 MAPK-, Erk1/2- and STAT1/STAT3-dependent pathways and is attenuated by interferon-γ. Basic Res. Cardiol..

[178] Nguyen M.T., Prima M.J., Song J.-A., Kim J., Do B.H., Yoo J., Park S., Jang J., Lee S., Lee E. (2019). Prokaryotic soluble overexpression and purification of oncostatin M using a fusion approach and genetically engineered *E. coli* strains. Sci. Rep..

[179] White U.A., Stewart W.C., Stephens J.M. (2011). Gp130 cytokines exert differential patterns of crosstalk in adipocytes both in vitro and in vivo. Obesity.

[180] Lörchner H., Pöling J., Gajawada P., Hou Y., Polyakova V., Kostin S., Adrian-Segarra J.M., Boettger T., Wietelmann A., Warnecke H. (2015). Myocardial healing requires Reg3β-dependent accumulation of macrophages in the ischemic heart. Nat. Med..

[181] Luo P., Wang P.-X., Li Z.-Z., Zhang X.-J., Jiang X., Gong J., Qin J.-J., Guo J., Zhu X., Yang S. (2016). Hepatic Oncostatin M Receptor β Regulates Obesity-Induced Steatosis and Insulin Resistance. Am. J. Pathol..

[182] Richards C.D. (2013). The Enigmatic Cytokine Oncostatin M and Roles in Disease. ISRN Inflamm..

[183] Zhang X., Li J., Qin J.-J., Cheng W.-L., Zhu X., Gong F.-H., She Z., Huang Z., Xia H., Li H. (2017). Oncostatin M receptor β deficiency attenuates atherogenesis by inhibiting JAK2/STAT3 signaling in macrophages. J. Lipid Res..

[184] Komori T., Morikawa Y. (2018). Oncostatin M in the development of metabolic syndrome and its potential as a novel therapeutic target. Anat. Sci. Int..

[185] Schnittker D., Kwofie K., Ashkar A., Trigatti B., Richards C.D. (2013). Oncostatin M and TLR-4 Ligand Synergize to Induce MCP-1, IL-6, and VEGF in Human Aortic Adventitial Fibroblasts and Smooth Muscle Cells. Mediat. Inflamm..

[186] Rychli K., Kaun C., Hohensinner P.J., Rega G., Pfaffenberger S., Vyskocil E., Breuss J.M., Furnkranz A., Uhrin P., Zaujec J. (2010). The inflammatory mediator oncostatin M induces angiopoietin 2 expression in endothelial cells in vitro and in vivo. J. Thromb. Haemost..

[187] Martini E., Kunderfranco P., Peano C., Carullo P., Cremonesi M., Schorn T., Carriero R., Termanini A., Colombo F.S., Jachetti E. (2019). Single-Cell Sequencing of Mouse Heart Immune Infiltrate in Pressure Overload-Driven Heart Failure Reveals Extent of Immune Activation. Circulation.

[188] Pöling J., Gajawada P., Richter M., Lörchner H., Polyakova V., Kostin S., Shin J., Boettger T., Walther T., Rees W. (2014). Therapeutic targeting of the oncostatin M receptor-β prevents inflammatory heart failure. Basic Res. Cardiol..

[189] Frangogiannis N.G. (2015). The reparative function of cardiomyocytes in the infarcted myocardium. Cell Metab..

[190] Setiadi H., Yago T., Liu Z., McEver R.P. (2019). Endothelial signaling by neutrophil-released oncostatin M enhances P-selectin–dependent inflammation and thrombosis. Blood Adv..

[191] Kirchmer M.N., Franco A., Albasanz-Puig A., Murray J., Yagi M., Gao L., Dong Z.M., Wijelath E.S. (2014). Modulation of vascular smooth muscle cell phenotype by STAT-1 and STAT-3. Atherosclerosis.

[192] Kakutani Y., Shioi A., Shoji T., Okazaki H., Koyama H., Emoto M., Inaba M. (2015). Oncostatin M Promotes Osteoblastic Differentiation of Human Vascular Smooth Muscle Cells Through JAK3-STAT3 Pathway. J. Cell. Biochem..

[193] Shioi A., Ikari Y. (2018). Plaque calcification during atherosclerosis progression and regression. J. Atheroscler. Thromb..

[194] Ghadge A.A., Khaire A.A., Kuvalekar A.A. (2018). Adiponectin: A potential therapeutic target for metabolic syndrome. Cytokine Growth Factor Rev..

[195] Oh D.K., Ciaraldi T., Henry R.R. (2007). Adiponectin in health and disease. Diabetes Obes. Metab..

[196] Harwood H.J., Heal D.J., Smith S.L., Jones R.B. (2012). The adipocyte as an endocrine organ in the regulation of metabolic homeostasis. Neuropharmacology.

[197] Jackson M.B., Ahima R.S. (2006). Neuroendocrine and metabolic effects of adipocyte-derived hormones. Clin. Sci..

[198] Chou I.-P., Chiu Y.-P., Ding S.-T., Liu B.-H., Lin Y.Y., Chen C.-Y. (2014). Adiponectin receptor 1 overexpression reduces lipid accumulation and hypertrophy in the heart of diet-induced obese mice—Possible involvement of oxidative stress and autophagy. Endocr. Res..

[199] Koentges C., König A., Pfeil K., Hölscher M.E., Schnick T., Wende A.R., Schrepper A., Cimolai M.C., Kersting S., Hoffmann M.M. (2015). Myocardial mitochondrial dysfunction in mice lacking adiponectin receptor 1. Basic Res. Cardiol..

[200] Braun M., Hettinger N., Koentges C., Pfeil K., Cimolai M.C., Hoffmann M.M., Osterholt M., Doenst T., Bode C., Bugger H. (2015). Myocardial mitochondrial and contractile function are preserved in mice lacking adiponectin. PLoS ONE.

[201] Berg A.H., Combs T.P., Scherer P.E. (2002). ACRP30/adiponectin: An adipokine regulating glucose and lipid metabolism. Trends Endocrinol. Metab..

[202] Hui X., Lam K.S.L., Vanhoutte P.M., Xu A. (2012). Adiponectin and cardiovascular health: An update. Br. J. Pharmacol..

[203] Ouchi N., Ohishi M., Kihara S., Funahashi T., Nakamura T., Nagaretani H., Kumada M., Ohashi K., Okamoto Y., Nishizawa H. (2003). Association of hypoadiponectinemia with impaired vasoreactivity. Hypertension.

[204] Zhang J., Xia L., Zhang F., Zhu D., Xin C., Wang H., Zhang F., Guo X., Lee Y., Zhang L. (2017). A novel mechanism of diabetic vascular endothelial dysfunction: Hypoadiponectinemia-induced NLRP3 inflammasome activation. Biochim. Biophys. Acta Mol. Basis Dis..

[205] Iwashima Y., Katsuya T., Ishikawa K., Ouchi N., Ohishi M., Sugimoto K., Fu Y., Motone M., Yamamoto K., Matsuo A. (2004). Hypoadiponectinemia Is an Independent Risk Factor for Hypertension. Hypertension.

[206] Chow W.-S., Cheung B.M.Y., Tso A.W.K., Xu A., Wat N.M.S., Fong C.H.Y., Ong L.H.Y., Tam S., Tan K.C.B., Janus E.D. (2007). Hypoadiponectinemia as a Predictor for the Development of Hypertension. Hypertension.

[207] Hashimoto N., Kanda J., Nakamura T., Horie A., Kurosawa H., Hashimoto T., Sato K., Kushida S., Suzuki M., Yano S. (2006). Association of hypoadiponectinemia in men with early onset of coronary heart disease and multiple coronary artery stenoses. Metabolism.

[208] Kumada M., Kihara S., Sumitsuji S., Kawamoto T., Matsumoto S., Ouchi N., Arita Y., Okamoto Y., Shimomura I., Hiraoka H. (2003). Association of Hypoadiponectinemia With Coronary Artery Disease in Men. Arterioscler. Thromb. Vasc. Biol..

[209] Mohty D., Pibarot P., Côté N., Cartier A., Audet A., Després J.P., Mathieu P. (2011). Hypoadiponectinemia Is Associated with Valvular Inflammation and Faster Disease Progression in Patients with Aortic Stenosis. Cardiology.

[210] Yanai H., Yoshida H. (2019). Beneficial Effects of Adiponectin on Glucose and Lipid Metabolism and Atherosclerotic Progression: Mechanisms and Perspectives. Int. J. Mol. Sci..

[211] Devaraj S., Torok N., Dasu M.R., Samols D., Jialal I. (2008). Adiponectin Decreases C-Reactive Protein Synthesis and Secretion from Endothelial Cells. Arterioscler. Thromb. Vasc. Biol..

[212] Zhou Y., Wei Y., Wang L., Wang X., Du X., Sun Z., Dong N., Chen X. (2011). Decreased adiponectin and increased inflammation expression in epicardial adipose tissue in coronary artery disease. Cardiovasc. Diabetol..

[213] Hattori Y., Nakano Y., Hattori S., Tomizawa A., Inukai K., Kasai K. (2008). High molecular weight adiponectin activates AMPK and suppresses cytokine-induced NF-κB activation in vascular endothelial cells. FEBS Lett..

[214] Wu X., Mahadev K., Fuchsel L., Ouedraogo R., Xu S.-Q., Goldstein B.J. (2007). Adiponectin suppresses IκB kinase activation induced by tumor necrosis factor-α or high glucose in endothelial cells: Role of cAMP and AMP kinase signaling. Am. J. Physiol. Metab..

[215] Ouedraogo R., Gong Y., Berzins B., Wu X., Mahadev K., Hough K., Chan L., Goldstein B.J., Scalia R. (2007). Adiponectin deficiency increases leukocyte-endothelium interactions via upregulation of endothelial cell adhesion molecules in vivo. J. Clin. Invest..

[216] Zhang H., Park Y., Zhang C. (2010). Coronary and Aortic Endothelial Function Affected by Feedback Between Adiponectin and Tumor Necrosis Factor α in Type 2 Diabetic Mice. Arterioscler. Thromb. Vasc. Biol..

[217] Shimabukuro M., Higa N., Asahi T., Oshiro Y., Takasu N., Tagawa T., Ueda S., Shimomura I., Funahashi T., Matsuzawa Y. (2003). Hypoadiponectinemia Is Closely Linked to Endothelial Dysfunction in Man. J. Clin. Endocrinol. Metab..

[218] Cao Y., Tao L., Yuan Y., Jiao X., Lau W.B., Wang Y., Christopher T., Lopez B., Chan L., Goldstein B. (2009). Endothelial dysfunction in adiponectin deficiency and its mechanisms involved. J. Mol. Cell. Cardiol..

[219] Deng G., Long Y., Yu Y.-R., Li M.-R. (2010). Adiponectin directly improves endothelial dysfunction in obese rats through the AMPK-eNOS Pathway. Int. J. Obes..

[220] Kelley N., Jeltema D., Duan Y., He Y. (2019). The NLRP3 Inflammasome: An Overview of Mechanisms of Activation and Regulation. Int. J. Mol. Sci..

[221] Van de Voorde J., Pauwels B., Boydens C., Decaluwé K. (2013). Adipocytokines in relation to cardiovascular disease. Metabolism.

[222] Piñeiro R., Iglesias M.J., Gallego R., Raghay K., Eiras S., Rubio J., Diéguez C., Gualillo O., González-Juanatey J.R., Lago F. (2005). Adiponectin is synthesized and secreted by human and murine cardiomyocytes. FEBS Lett..

[223] Boddu N.J., Theus S., Luo S., Wei J.Y., Ranganathan G. (2014). Is the lack of adiponectin associated with increased ER/SR stress and inflammation in the heart?. Adipocyte.

[224] Hou Y., Wang X.F., Lang Z.Q., Jin Y.C., Fu J.R., Xv X.M., Sun S.T., Xin X., Zhang L.S. (2018). Adiponectin is protective against endoplasmic reticulum stress-induced apoptosis of endothelial cells in sepsis. Braz. J. Med. Biol. Res..

[225] Ding W., Zhang X., Huang H., Ding N., Zhang S., Hutchinson S.Z., Zhang X. (2014). Adiponectin protects rat myocardium against chronic intermittent hypoxia-induced injury via inhibition of endoplasmic reticulum stress. PLoS ONE.

[226] Guo J., Bian Y., Bai R., Li H., Fu M., Xiao C. (2013). Globular adiponectin attenuates myocardial ischemia/reperfusion injury by upregulating endoplasmic reticulum Ca^2+^-ATPase activity and inhibiting endoplasmic reticulum stress. J. Cardiovasc. Pharmacol..

[227] Bian Y.-F., Hao X.-Y., Gao F., Yang H.-Y., Zang N., Xiao C.-S. (2011). Adiponectin attenuates hypoxia/reoxygenation-induced cardiomyocyte injury through inhibition of endoplasmic reticulum stress. J. Investig. Med..

[228] Gao C., Liu Y., Yu Q., Yang Q., Li B., Sun L., Yan W., Cai X., Gao E., Xiong L. (2015). TNF-α antagonism ameliorates myocardial ischemia-reperfusion injury in mice by upregulating adiponectin. Am. J. Physiol. Heart Circ. Physiol..

[229] Oh-I S., Shimizu H., Satoh T., Okada S., Adachi S., Inoue K., Eguchi H., Yamamoto M., Imaki T., Hashimoto K. (2006). Identification of nesfatin-1 as a satiety molecule in the hypothalamus. Nature.

[230] Feijóo-Bandín S., Rodríguez-Penas D., García-Rúa V., Mosquera-Leal A., González-Juanatey J.R., Lago F. (2016). Nesfatin-1: A new energy-regulating peptide with pleiotropic functions. Implications at cardiovascular level. Endocrine.

[231] Ramanjaneya M., Chen J., Brown J.E., Tripathi G., Hallschmid M., Patel S., Kern W., Hillhouse E.W., Lehnert H., Tan B.K. (2010). Identification of nesfatin-1 in human and murine adipose tissue: A novel depot-specific adipokine with increased levels in obesity. Endocrinology.

[232] St-Pierre D.H., Martin J., Shimizu H., Tagaya Y., Tsuchiya T., Marceau S., Biertho L., Bastien M., Caron-Cantin S.-M., Simard S. (2016). Association between nesfatin-1 levels and metabolic improvements in severely obese patients who underwent biliopancreatic derivation with duodenal switch. Peptides.

[233] Gonzalez R., Reingold B.K., Gao X., Gaidhu M.P., Tsushima R.G., Unniappan S. (2011). Nesfatin-1 exerts a direct, glucose-dependent insulinotropic action on mouse islet β- and MIN6 cells. J. Endocrinol..

[234] Bonnet M.S., Pecchi E., Trouslard J., Jean A., Dallaporta M., Troadec J.-D. (2009). Central nesfatin-1-expressing neurons are sensitive to peripheral inflammatory stimulus. J. Neuroinflamm..

[235] Jiang L., Xu K., Li J., Zhou X., Xu L., Wu Z., Ma C., Ran J., Hu P., Bao J. (2020). Nesfatin-1 suppresses interleukin-1β-induced inflammation, apoptosis, and cartilage matrix destruction in chondrocytes and ameliorates osteoarthritis in rats. Aging.

[236] Wang Z.-Z., Chen S.-C., Zou X.-B., Tian L.-L., Sui S.-H., Liu N.-Z. (2020). Nesfatin-1 alleviates acute lung injury through reducing inflammation and oxidative stress via the regulation of HMGB1. Eur. Rev. Med. Pharmacol. Sci..

[237] Tang C.-H., Fu X.-J., Xu X.-L., Wei X.-J., Pan H.-S. (2012). The anti-inflammatory and anti-apoptotic effects of nesfatin-1 in the traumatic rat brain. Peptides.

[238] Özsavcí D., Erşahin M., Şener A., Özakpinar Ö.B., Toklu H.Z., Akakín D., Şener G., Yeğen B.Ç. (2011). The novel function of nesfatin-1 as an anti-inflammatory and antiapoptotic peptide in subarachnoid hemorrhage-induced oxidative brain damage in rats. Neurosurgery.

[239] Acik V., Matyar S., Arslan A., İstemen İ., Olguner S.K., Arslan B., Gezercan Y., Ökten A.İ. (2020). Relationshıp of spontaneous subarachnoid haemorrhage and cerebral aneurysm to serum Visfatin and Nesfatin-1 levels. Clin. Neurol. Neurosurg..

[240] Feijóo-Bandín S., Rodríguez-Penas D., García-Rúa V., Mosquera-Leal A., Otero M.F., Pereira E., Rubio J., Martínez I., Seoane L.M., Gualillo O. (2013). Nesfatin-1 in human and murine cardiomyocytes: Synthesis, secretion, and mobilization of GLUT-4. Endocrinology.

[241] Ibe S., Kishimoto Y., Niki H., Saita E., Umei T., Miura K., Ikegami Y., Ohmori R., Kondo K., Momiyama Y. (2019). Associations between plasma nesfatin-1 levels and the presence and severity of coronary artery disease. Heart Vessels.

[242] Serdar Kuyumcu M., Kuyumcu A., Yayla Ç., Bilal Özbay M., Ünal S., Açar B., Nural C., Şenat A., Samur G. (2018). The Relationship between Nesfatin-1 Levels and SYNTAX Score in Patients with Non-ST Segment Elevation Myocardial Infarction. Acta Cardiol. Sin..

[243] Kuyumcu A., Kuyumcu M.S., Ozbay M.B., Ertem A.G., Samur G. (2019). Nesfatin-1: A novel regulatory peptide associated with acute myocardial infarction and Mediterranean diet. Peptides.

[244] Dai H., Li X., He T., Wang Y., Wang Z., Wang S., Xing M., Sun W., Ding H. (2013). Decreased plasma nesfatin-1 levels in patients with acute myocardial infarction. Peptides.

[245] Sivri S., Sökmen E., Çelik M., Güçlü K. (2020). Nesfatin-1 Levels Predict Angiographic No-Reflow in Patients with ST-Segment Elevation Myocardial Infarction. Acta Cardiol. Sin..

[246] Kirisci M., Yardimci M.M., Kocarslan A., Sokmen A., Doganer A., Gunes H. (2020). Nesfatin 1: A promising biomarker predicting successful reperfusion after coronary artery bypass surgery. Bratisl. Med. J..

[247] Tasatargil A., Kuscu N., Dalaklioglu S., Adiguzel D., Celik-Ozenci C., Ozdem S., Barutcigil A., Ozdem S. (2017). Cardioprotective effect of nesfatin-1 against isoproterenol-induced myocardial infarction in rats: Role of the Akt/GSK-3β pathway. Peptides.

[248] Naseroleslami M., Sharifi M., Rakhshan K., Mokhtari B., Aboutaleb N. (2020). Nesfatin-1 attenuates injury in a rat model of myocardial infarction by targeting autophagy, inflammation, and apoptosis. Arch. Physiol. Biochem..

[249] Feijóo-Bandín S., Aragón-Herrera A., Rodríguez-Penas D., Portolés M., Roselló-Lletí E., Rivera M., González-Juanatey J.R., Lago F. (2017). Relaxin-2 in Cardiometabolic Diseases: Mechanisms of Action and Future Perspectives. Front. Physiol..

[250] Olefsky J.M., Saekow M., Kroc R.L. (1982). Potentiation of insulin binding and insulin action by purified porcine relaxin. Ann. N. Y. Acad. Sci..

[251] Jarrett J.C., Ballejo G., Saleem T.H., Tsibris J.C.M., Spellacy W.N. (1984). The effect of prolactin and relaxin on insulin binding by adipocytes from pregnant women. Am. J. Obstet. Gynecol..

[252] Bani G., Bianchi S., Formigli L., Bigazzi M. (1989). Responsiveness of Mouse Parametrial Fat to Relaxin. Cells Tissues Organs.

[253] Martin B., Romero G., Salama G. (2019). Cardioprotective actions of relaxin. Mol. Cell. Endocrinol..

[254] Masini E., Bani D., Bigazzi M., Mannaioni P.F., Bani-Sacchi T. (1994). Effects of relaxin on mast cells. In vitro and in vivo studies in rats and guinea pigs. J. Clin. Invest..

[255] Bani D., Bigazzi M., Masini E., Bani G., Sacchi T.B. (1995). Relaxin depresses platelet aggregation: In vitro studies on isolated human and rabbit platelets. Lab. Invest..

[256] Bani D., Masini E., Bello M.G., Bigazzi M., Bani Sacchi T., Sacchi T.B. (1998). Relaxin protects against myocardial injury caused by ischemia and reperfusion in rat heart. Am. J. Pathol..

[257] Masini E., Bani D., Bello M.G., Bigazzi M., Mannaioni P.F., Sacchi T.B. (1997). Relaxin counteracts myocardial damage induced by ischemia-reperfusion in isolated guinea pig hearts: Evidence for an involvement of nitric oxide. Endocrinology.

[258] Nistri S., Cinci L., Perna A.M., Masini E., Mastroianni R., Bani D. (2008). Relaxin induces mast cell inhibition and reduces ventricular arrhythmias in a swine model of acute myocardial infarction. Pharmacol. Res..

[259] Nistri S., Chiappini L., Sassoli C., Bani D. (2003). Relaxin inhibits lipopolysaccharide-induced adhesion of neutrophils to coronary endothelial cells by a nitric oxide-mediated mechanism. FASEB J..

[260] Masini E., Nistri S., Vannacci A., Bani Sacchi T., Novelli A., Bani D. (2004). Relaxin Inhibits the Activation of Human Neutrophils: Involvement of the Nitric Oxide Pathway. Endocrinology.

[261] Valle Raleigh J., Mauro A.G., Devarakonda T., Marchetti C., He J., Kim E., Filippone S., Das A., Toldo S., Abbate A. (2017). Reperfusion therapy with recombinant human relaxin-2 (Serelaxin) attenuates myocardial infarct size and NLRP3 inflammasome following ischemia/reperfusion injury via eNOS-dependent mechanism. Cardiovasc. Res..

[262] Toldo S., Mauro A.G., Cutter Z., Abbate A. (2018). Inflammasome, pyroptosis, and cytokines in myocardial ischemia-reperfusion injury. Am. J. Physiol. Circ. Physiol..

[263] Beiert T., Tiyerili V., Knappe V., Effelsberg V., Linhart M., Stöckigt F., Klein S., Schierwagen R., Trebicka J., Nickenig G. (2017). Relaxin reduces susceptibility to post-infarct atrial fibrillation in mice due to anti-fibrotic and anti-inflammatory properties. Biochem. Biophys. Res. Commun..

[264] Beiert T., Knappe V., Tiyerili V., Stöckigt F., Effelsberg V., Linhart M., Steinmetz M., Klein S., Schierwagen R., Trebicka J. (2018). Chronic lower-dose relaxin administration protects from arrhythmia in experimental myocardial infarction due to anti-inflammatory and anti-fibrotic properties. Int. J. Cardiol..

[265] Sanchez-Mas J., Lax A., Asensio-Lopez M.C., Lencina M., Fernandez-del Palacio M.J., Soriano-Filiu A., de Boer R.A., Pascual-Figal D.A. (2017). Early Anti-inflammatory and Pro-angiogenic Myocardial Effects of Intravenous Serelaxin Infusion for 72 H in an Experimental Rat Model of Acute Myocardial Infarction. J. Cardiovasc. Transl. Res..

[266] Gao X.-M., Su Y., Moore S., Han L.-P., Kiriazis H., Lu Q., Zhao W.-B., Ruze A., Fang B.-B., Duan M.-J. (2019). Relaxin mitigates microvascular damage and inflammation following cardiac ischemia—Reperfusion. Basic Res. Cardiol..

[267] Van den Berg N.W.E., de Groot J.R. (2015). Myocardial infarction, atrial fibrillation and mortality: Timing is everything. Neth. Heart. J..

[268] Samuel C.S., Royce S.G., Hewitson T.D., Denton K.M., Cooney T.E., Bennett R.G. (2017). Anti-fibrotic actions of relaxin. Br. J. Pharmacol..

[269] Wang D., Zhu H., Yang Q., Sun Y. (2016). Effects of relaxin on cardiac fibrosis, apoptosis, and tachyarrhythmia in rats with myocardial infarction. Biomed. Pharmacother..

[270] Martin B., Gabris-Weber B.A., Reddy R., Romero G., Chattopadhyay A., Salama G. (2018). Relaxin reverses inflammatory and immune signals in aged hearts. PLoS ONE.

[271] Brecht A., Bartsch C., Baumann G., Stangl K., Dschietzig T. (2011). Relaxin inhibits early steps in vascular inflammation. Regul. Pept..

[272] Dschietzig T., Bartsch C., Baumann G., Stangl K. (2009). RXFP1-inactive relaxin activates human glucocorticoid receptor: Further investigations into the relaxin-GR pathway. Regul. Pept..

[273] Bathgate R.A.D., Halls M.L., van der Westhuizen E.T., Callander G.E., Kocan M., Summers R.J. (2013). Relaxin family peptides and their receptors. Physiol. Rev..

[274] Collino M., Rogazzo M., Pini A., Benetti E., Rosa A.C., Chiazza F., Fantozzi R., Bani D., Masini E. (2013). Acute treatment with relaxin protects the kidney against ischaemia/reperfusion injury. J. Cell. Mol. Med..

[275] Boehnert M.U., Armbruster F.P., Hilbig H. (2009). Relaxin as a protective substance in the preserving solution for liver transplantation: Spectrophotometric in vivo imaging of local oxygen supply in an isolated perfused rat liver model. Ann. N. Y. Acad. Sci..

[276] Komiya T., Tanigawa Y., Hirohashi S. (1998). Cloning of the Novel Gene Intelectin, Which Is Expressed in Intestinal Paneth Cells in Mice. Biochem. Biophys. Res. Commun..

[277] Suzuki Y.A., Shin K., Lönnerdal B. (2001). Molecular Cloning and Functional Expression of a Human Intestinal Lactoferrin Receptor ‡. Biochemistry.

[278] Lee J.-K., Schnee J., Pang M., Wolfert M., Baum L.G., Moremen K.W., Pierce M. (2001). Human homologs of the Xenopus oocyte cortical granule lectin XL35. Glycobiology.

[279] Schäffler A., Neumeier M., Herfarth H., Fürst A., Schölmerich J., Büchler C. (2005). Genomic structure of human omentin, a new adipocytokine expressed in omental adipose tissue. Biochim. Biophys. Acta Gene Struct. Expr..

[280] Yang R.-Z., Lee M.-J., Hu H., Pray J., Wu H.-B., Hansen B.C., Shuldiner A.R., Fried S.K., McLenithan J.C., Gong D.-W. (2006). Identification of omentin as a novel depot-specific adipokine in human adipose tissue: Possible role in modulating insulin action. Am. J. Physiol. Metab..

[281] Fain J.N., Sacks H.S., Buehrer B., Bahouth S.W., Garrett E., Wolf R.Y., Carter R.A., Tichansky D.S., Madan A.K. (2008). Identification of omentin mRNA in human epicardial adipose tissue: Comparison to omentin in subcutaneous, internal mammary artery periadventitial and visceral abdominal depots. Int. J. Obes..

[282] Svensson H., Odén B., Edén S., Lönn M. (2014). Adiponectin, chemerin, cytokines, and dipeptidyl peptidase 4 are released from human adipose tissue in a depot-dependent manner: An in vitro system including human serum albumin. BMC Endocr. Disord..

[283] Du Y., Ji Q., Cai L., Huang F., Lai Y., Liu Y., Yu J., Han B., Zhu E., Zhang J. (2016). Association between omentin-1 expression in human epicardial adipose tissue and coronary atherosclerosis. Cardiovasc. Diabetol..

[284] Matloch Z., Kratochvílová H., Cinkajzlová A., Lipš M., Kopecký P., Pořízka M., Haluzíková D., Lindner J., Mráz M., Kloučková J. (2018). Changes in Omentin Levels and Its mRNA Expression in Epicardial Adipose Tissue in Patients Undergoing Elective Cardiac Surgery: The Influence of Type 2 Diabetes and Coronary Heart Disease. Physiol. Res..

[285] Harada K., Shibata R., Ouchi N., Tokuda Y., Funakubo H., Suzuki M., Kataoka T., Nagao T., Okumura S., Shinoda N. (2016). Increased expression of the adipocytokine omentin in the epicardial adipose tissue of coronary artery disease patients. Atherosclerosis.

[286] Berti L., Hartwig S., Irmler M., Rädle B., Siegel-Axel D., Beckers J., Lehr S., Al-Hasani H., Häring H.-U., Hrabě de Angelis M. (2016). Impact of fibroblast growth factor 21 on the secretome of human perivascular preadipocytes and adipocytes: A targeted proteomics approach. Arch. Physiol. Biochem..

[287] De Souza Batista C.M., Yang R.-Z., Lee M.-J., Glynn N.M., Yu D.-Z., Pray J., Ndubuizu K., Patil S., Schwartz A., Kligman M. (2007). Omentin Plasma Levels and Gene Expression Are Decreased in Obesity. Diabetes.

[288] Fernández-Trasancos Á., Agra R.M., García-Acuña J.M., Fernández Á.L., González-Juanatey J.R., Eiras S. (2017). Omentin treatment of epicardial fat improves its anti-inflammatory activity and paracrine benefit on smooth muscle cells. Obesity.

[289] Zhou H., Zhang Z., Qian G., Zhou J. (2020). Omentin-1 attenuates adipose tissue inflammation via restoration of TXNIP/NLRP3 signaling in high-fat diet-induced obese mice. Fundam. Clin. Pharmacol..

[290] Wang J., Gao Y., Lin F., Han K., Wang X. (2020). Omentin-1 attenuates lipopolysaccharide (LPS)-induced U937 macrophages activation by inhibiting the TLR4/MyD88/NF-κB signaling. Arch. Biochem. Biophys..

[291] Yıldız S.S., Sahin I., Cetinkal G., Aksan G., Kucuk S.H., Keskin K., Cetin S., Sigirci S., Avcı İ.İ., Kilci H. (2018). Usefulness of Serum Omentin-1 Levels for the Prediction of Adverse Cardiac Events in Patients with Hypertrophic Cardiomyopathy. Med. Princ. Pract..

[292] Wang X.-H., Dou L.-Z., Gu C., Wang X.-Q. (2014). Plasma levels of omentin-1 and visfatin in senile patients with coronary heart disease and heart failure. Asian Pac. J. Trop. Med..

[293] Narumi T., Watanabe T., Kadowaki S., Kinoshita D., Yokoyama M., Honda Y., Otaki Y., Nishiyama S., Takahashi H., Arimoto T. (2014). Impact of serum omentin-1 levels on cardiac prognosis in patients with heart failure. Cardiovasc. Diabetol..

[294] Huang Y., Lin Y., Zhang S., Wang Z., Zhang J., Chang C., Liu L., Ji Q., Liu X. (2016). Circulating Omentin-1 Levels Are Decreased in Dilated Cardiomyopathy Patients with Overt Heart Failure. Dis. Markers.

[295] Biscetti F., Nardella E., Bonadia N., Angelini F., Pitocco D., Santoliquido A., Filipponi M., Landolfi R., Flex A. (2019). Association between plasma omentin-1 levels in type 2 diabetic patients and peripheral artery disease. Cardiovasc. Diabetol..

[296] Onur I., Oz F., Yildiz S., Kuplay H., Yucel C., Sigirci S., Elitok A., Pilten S., Kasali K., Yasar Cizgici A. (2014). A decreased serum omentin-1 level may be an independent risk factor for peripheral arterial disease. Int. Angiol..

[297] Baig M., Alghalayini K.W., Gazzaz Z.J., Atta H. (2020). Association of Serum Omentin-1, Chemerin, and Leptin with Acute Myocardial Infarction and its Risk Factors. Pakistan J. Med. Sci..

[298] Kadoglou N.P.E., Tahmatzidis D.K., Giannakoulas C., Kapelouzou A., Gkontopoulos A., Parissis J., Lampropoulos S., Kottas G. (2015). Serum levels of novel adipokines, omentin-1 and chemerin, in patients with acute myocardial infarction: KOZANI STUDY. J. Cardiovasc. Med..

[299] Chen Y., Liu F., Han F., Lv L., Tang C.-E., Luo F. (2020). Omentin-1 Ameliorated Free Fatty Acid-Induced Impairment in Proliferation, Migration, and Inflammatory States of HUVECs. Cardiol. Res. Pract..

[300] Çimen A.R., Cerit E.T., Iyidir O.T., Karakus R., Uyar B.B., Toruner F.B., Cakir N., Arslan M. (2017). Serum omentin-1 levels and endothelial dysfunction in obesity. Acta Endocrinol..

[301] Hayashi M., Morioka T., Hatamori M., Kakutani Y., Yamazaki Y., Kurajoh M., Motoyama K., Mori K., Fukumoto S., Shioi A. (2019). Plasma omentin levels are associated with vascular endothelial function in patients with type 2 diabetes at elevated cardiovascular risk. Diabetes Res. Clin. Pract..

[302] Moreno-Navarrete J.M., Ortega F., Castro A., Sabater M., Ricart W., Fernández-Real J.M. (2011). Circulating Omentin as a Novel Biomarker of Endothelial Dysfunction. Obesity.

[303] Liu F., Fang S., Liu X., Li J., Wang X., Cui J., Chen T., Li Z., Yang F., Tian J. (2020). Omentin-1 protects against high glucose-induced endothelial dysfunction via the AMPK/PPARδ signaling pathway. Biochem. Pharmacol..

[304] Yamawaki H., Kuramoto J., Kameshima S., Usui T., Okada M., Hara Y. (2011). Omentin, a novel adipocytokine inhibits TNF-induced vascular inflammation in human endothelial cells. Biochem. Biophys. Res. Commun..

[305] Zhong X., Li X., Liu F., Tan H., Shang D. (2012). Omentin inhibits TNF-α-induced expression of adhesion molecules in endothelial cells via ERK/NF-κB pathway. Biochem. Biophys. Res. Commun..

[306] Binti Kamaruddin N.A., Fong L.Y., Tan J.J., Abdullah M.N.H., Singh Cheema M., Bin Yakop F., Yong Y.K. (2020). Cytoprotective Role of Omentin Against Oxidative Stress-Induced Vascular Endothelial Cells Injury. Molecules.

[307] Luo L., Liu M. (2016). Adipose tissue in control of metabolism. J. Endocrinol..

[308] Ushach I., Arrevillaga-Boni G., Heller G.N., Pone E., Hernandez-Ruiz M., Catalan-Dibene J., Hevezi P., Zlotnik A. (2018). Meteorin-like/Meteorin-β Is a Novel Immunoregulatory Cytokine Associated with Inflammation. J. Immunol..

[309] Lee J.O., Byun W.S., Kang M.J., Han J.A., Moon J., Shin M.-J., Lee H.J., Chung J.H., Lee J.-S., Son C.-G. (2020). The myokine meteorin-like (metrnl) improves glucose tolerance in both skeletal muscle cells and mice by targeting AMPKα2. FEBS J..

[310] Rao R.R., Long J.Z., White J.P., Svensson K.J., Lou J., Lokurkar I., Jedrychowski M.P., Ruas J.L., Wrann C.D., Lo J.C. (2014). Meteorin-like is a hormone that regulates immune-adipose interactions to increase beige fat thermogenesis. Cell.

[311] Lee J.H., Kang Y.E., Kim J.M., Choung S., Joung K.H., Kim H.J., Ku B.J. (2018). Serum Meteorin-like protein levels decreased in patients newly diagnosed with type 2 diabetes. Diabetes Res. Clin. Pract..

[312] Dadmanesh M., Aghajani H., Fadaei R., Ghorban K. (2018). Lower serum levels of Meteorin-like/Subfatin in patients with coronary artery disease and type 2 diabetes mellitus are negatively associated with insulin resistance and inflammatory cytokines. PLoS ONE.

[313] Liu Z.-X., Ji H.-H., Yao M.-P., Wang L., Wang Y., Zhou P., Liu Y., Zheng X.-F., He H.-W., Wang L.-S. (2019). Serum Metrnl is associated with the presence and severity of coronary artery disease. J. Cell. Mol. Med..

[314] El-Ashmawy H.M., Selim F.O., Hosny T.A.M., Almassry H.N. (2019). Association of low serum Meteorin like (Metrnl) concentrations with worsening of glucose tolerance, impaired endothelial function and atherosclerosis. Diabetes Res. Clin. Pract..

[315] Xu L., Cai Y., Wang Y., Xu C. (2020). Meteorin-Like (METRNL) Attenuates Myocardial Ischemia/Reperfusion Injury-Induced Cardiomyocytes Apoptosis by Alleviating Endoplasmic Reticulum Stress via Activation of AMPK-PAK2 Signaling in H9C2 Cells. Med. Sci. Monit..

[316] Fisher F.M., Maratos-Flier E. (2016). Understanding the Physiology of FGF21. Annu. Rev. Physiol..

[317] Haberka M., Machnik G., Kowalówka A., Biedroń M., Skudrzyk E., Regulska-Ilow B., Gajos G., Manka R., Deja M., Okopień B. (2019). Epicardial, paracardial and perivascular fat quantity, genes expression and serum cytokines in coronary artery disease and diabetes. Pol. Arch. Intern. Med..

[318] Akyildiz Z.I., Polat S., Yurekli B.S., Kocabas G.U., Tuluce K., Tuluce S.Y., Kocabas U., Bozkaya G., Yuksel A., Nazli C. (2015). Epicardial fat, body mass index, and triglyceride are independent contributors of serum fibroblast growth factor 21 level in obese premenopausal women. J. Endocrinol. Invest..

[319] Kotulák T., Drápalová J., Kopecký P., Lacinová Z., Kramář P., Říha H., Netuka I., Malý J., Housa D., Bláha J. (2011). Increased circulating and epicardial adipose tissue mRNA expression of fibroblast growth factor-21 after cardiac surgery: Possible role in postoperative inflammatory response and insulin resistance. Physiol. Res..

[320] Wang W.-F., Li S.-M., Ren G.-P., Zheng W., Lu Y.-J., Yu Y.-H., Xu W.-J., Li T.-H., Zhou L.-H., Liu Y. (2015). Recombinant murine fibroblast growth factor 21 ameliorates obesity-related inflammation in monosodium glutamate-induced obesity rats. Endocrine.

[321] Wang N., Zhao T.-T., Li S., Sun X., Li Z., Li Y., Li D.-S., Wang W.-F. (2019). Fibroblast Growth Factor 21 Exerts its Anti-inflammatory Effects on Multiple Cell Types of Adipose Tissue in Obesity. Obesity.

[322] Keinicke H., Sun G., Mentzel C.M.J., Fredholm M., John L.M., Andersen B., Raun K., Kjaergaard M. (2020). FGF21 regulates hepatic metabolic pathways to improve steatosis and inflammation. Endocr. Connect..

[323] Singhal G., Fisher F.M., Chee M.J., Tan T.G., El Ouaamari A., Adams A.C., Najarian R., Kulkarni R.N., Benoist C., Flier J.S. (2016). Fibroblast Growth Factor 21 (FGF21) Protects against High Fat Diet Induced Inflammation and Islet Hyperplasia in Pancreas. PLoS ONE.

[324] Gao J., Liu Q., Li J., Hu C., Zhao W., Ma W., Yao M., Xing L. (2020). Fibroblast Growth Factor 21 dependent TLR4/MYD88/NF-κB signaling activation is involved in lipopolysaccharide-induced acute lung injury. Int. Immunopharmacol..

[325] Zhang J., Cheng Y., Gu J., Wang S., Zhou S., Wang Y., Tan Y., Feng W., Fu Y., Mellen N. (2016). Fenofibrate increases cardiac autophagy via FGF21/SIRT1 and prevents fibrosis and inflammation in the hearts of Type 1 diabetic mice. Clin. Sci..

[326] Jung J.G., Yi S.-A., Choi S.-E., Kang Y., Kim T.H., Jeon J.Y., Bae M.A., Ahn J.H., Jeong H., Hwang E.S. (2015). TM-25659-Induced Activation of FGF21 Level Decreases Insulin Resistance and Inflammation in Skeletal Muscle via GCN2 Pathways. Mol. Cells.

[327] Wang N., Li J.-Y., Li S., Guo X.-C., Wu T., Wang W.-F., Li D.-S. (2018). Fibroblast growth factor 21 regulates foam cells formation and inflammatory response in Ox-LDL-induced THP-1 macrophages. Biomed. Pharmacother..

[328] Yu Y., He J., Li S., Song L., Guo X., Yao W., Zou D., Gao X., Liu Y., Bai F. (2016). Fibroblast growth factor 21 (FGF21) inhibits macrophage-mediated inflammation by activating Nrf2 and suppressing the NF-κB signaling pathway. Int. Immunopharmacol..

[329] Zeng Z., Zheng Q., Chen J., Tan X., Li Q., Ding L., Zhang R., Lin X. (2020). FGF21 mitigates atherosclerosis via inhibition of NLRP3 inflammasome-mediated vascular endothelial cells pyroptosis. Exp. Cell Res..

[330] Yan X., Gou Z., Li Y., Wang Y., Zhu J., Xu G., Zhang Q. (2018). Fibroblast growth factor 21 inhibits atherosclerosis in apoE-/- mice by ameliorating Fas-mediated apoptosis. Lipids Health Dis..

[331] Xiaolong L., Dongmin G., Liu M., Zuo W., Huijun H., Qiufen T., XueMei H., Wensheng L., Yuping P., Jun L. (2020). FGF21 induces autophagy-mediated cholesterol efflux to inhibit atherogenesis via RACK1 up-regulation. J. Cell. Mol. Med..

[332] Wu X., Qi Y.-F., Chang J.-R., Lu W.-W., Zhang J.-S., Wang S.-P., Cheng S.-J., Zhang M., Fan Q., Lv Y. (2015). Possible role of fibroblast growth factor 21 on atherosclerosis via amelioration of endoplasmic reticulum stress-mediated apoptosis in apoE(-/-) mice. Heart Vessels.

[333] Lin Z., Pan X., Wu F., Ye D., Zhang Y., Wang Y., Jin L., Lian Q., Huang Y., Ding H. (2015). Fibroblast Growth Factor 21 Prevents Atherosclerosis by Suppression of Hepatic Sterol Regulatory Element-Binding Protein-2 and Induction of Adiponectin in Mice. Circulation.

[334] Jia H., Cheng J., Zhou Q., Peng J., Pan Y., Han H. (2018). Fibroblast growth factor 21 attenuates inflammation and oxidative stress in atherosclerotic rat via enhancing the Nrf1-ARE signaling pathway. Int. J. Clin. Exp. Pathol..

[335] Zhang Y., Liu Z., Zhou M., Liu C. (2018). Therapeutic effects of fibroblast growth factor-21 against atherosclerosis via the NF-κB pathway. Mol. Med. Rep..

[336] Ying L., Li N., He Z., Zeng X., Nan Y., Chen J., Miao P., Ying Y., Lin W., Zhao X. (2019). Fibroblast growth factor 21 Ameliorates diabetes-induced endothelial dysfunction in mouse aorta via activation of the CaMKK2/AMPKα signaling pathway. Cell Death Dis..

[337] Basurto L., Gregory M.A., Hernández S.B., Sánchez-Huerta L., Martínez A.D., Manuel-Apolinar L., Avelar F.J., Alonso L.A.M., Sánchez-Arenas R. (2019). Monocyte chemoattractant protein-1 (MCP-1) and fibroblast growth factor-21 (FGF-21) as biomarkers of subclinical atherosclerosis in women. Exp. Gerontol..

[338] Yafei S., Elsewy F., Youssef E., Ayman M., El-Shafei M. (2019). Fibroblast growth factor 21 association with subclinical atherosclerosis and arterial stiffness in type 2 diabetes. Diabetes Metab. Syndr..

[339] Wu L., Qian L., Zhang L., Zhang J., Zhou J., Li Y., Hou X., Fang Q., Li H., Jia W. (2020). Fibroblast Growth Factor 21 is Related to Atherosclerosis Independent of Nonalcoholic Fatty Liver Disease and Predicts Atherosclerotic Cardiovascular Events. J. Am. Heart Assoc..

[340] Wang X., Huang X., Hou J. (2016). Relationship between Serum fibroblast growth factor 21 levels and morphological atherosclerotic plaque characteristics in patients with coronary heart disease. Eur. Heart J. Suppl..

[341] Xiao Y., Liu L., Xu A., Zhou P., Long Z., Tu Y., Chen X., Tang W., Huang G., Zhou Z. (2015). Serum fibroblast growth factor 21 levels are related to subclinical atherosclerosis in patients with type 2 diabetes. Cardiovasc. Diabetol..

[342] Miyazaki Y., Saita E., Kishimoto Y., Ibe S., Seki T., Miura K., Suzuki-Sugihara N., Ikegami Y., Ohmori R., Kondo K. (2018). Low Plasma Levels of Fibroblast Growth Factor-21 in Patients with Peripheral Artery Disease. J. Atheroscler. Thromb..

[343] Yang H., Feng A., Lin S., Yu L., Lin X., Yan X., Lu X., Zhang C. (2018). Fibroblast growth factor-21 prevents diabetic cardiomyopathy via AMPK-mediated antioxidation and lipid-lowering effects in the heart. Cell Death Dis..

[344] Tanajak P., Sa-Nguanmoo P., Wang X., Liang G., Li X., Jiang C., Chattipakorn S.C., Chattipakorn N. (2016). Fibroblast growth factor 21 (FGF21) therapy attenuates left ventricular dysfunction and metabolic disturbance by improving FGF21 sensitivity, cardiac mitochondrial redox homoeostasis and structural changes in pre-diabetic rats. Acta Physiol..

[345] Li S., Zhu Z., Xue M., Yi X., Liang J., Niu C., Chen G., Shen Y., Zhang H., Zheng J. (2019). Fibroblast growth factor 21 protects the heart from angiotensin II-induced cardiac hypertrophy and dysfunction via SIRT1. Biochim. Biophys. Acta Mol. Basis Dis..

[346] Planavila A., Redondo-Angulo I., Ribas F., Garrabou G., Casademont J., Giralt M., Villarroya F. (2015). Fibroblast growth factor 21 protects the heart from oxidative stress. Cardiovasc. Res..

[347] Planavila A., Redondo I., Hondares E., Vinciguerra M., Munts C., Iglesias R., Gabrielli L.A., Sitges M., Giralt M., van Bilsen M. (2013). Fibroblast growth factor 21 protects against cardiac hypertrophy in mice. Nat. Commun..

[348] Li J., Xu C., Liu Y., Li Y., Du S., Zhang R., Sun Y., Zhang R., Wang Y., Xue H. (2020). Fibroblast growth factor 21 inhibited ischemic arrhythmias via targeting miR-143/EGR1 axis. Basic Res. Cardiol..

